# Deep sequencing and SNP array analyses of pediatric T-cell acute lymphoblastic leukemia reveal *NOTCH1* mutations in minor subclones and a high incidence of uniparental isodisomies affecting *CDKN2A*

**DOI:** 10.1186/s13045-015-0138-0

**Published:** 2015-04-24

**Authors:** Kristina Karrman, Anders Castor, Mikael Behrendtz, Erik Forestier, Linda Olsson, Mats Ehinger, Andrea Biloglav, Thoas Fioretos, Kajsa Paulsson, Bertil Johansson

**Affiliations:** Department of Clinical Genetics, University and Regional Laboratories, Region Skåne, SE-221 85 Lund, Sweden; Division of Clinical Genetics, Department of Laboratory Medicine, Lund University, Lund, Sweden; Department of Pediatrics, Skåne University Hospital, Lund University, Lund, Sweden; Department of Pediatrics, Linköping University Hospital, Linköping, Sweden; Department of Medical Biosciences, Clinical Genetics, Umeå University, Umeå, Sweden; Department of Pathology, University and Regional Laboratories, Region Skåne, Lund, Sweden

**Keywords:** T-ALL, Pediatric, Genetic characterization, SNP array, Large-scale sequencing

## Abstract

**Background:**

Pediatric T-cell acute lymphoblastic leukemia (T-ALL) is a genetically heterogeneous disease that arises in a multistep fashion through acquisition of several genetic aberrations, subsequently giving rise to a malignant, clonal expansion of T-lymphoblasts. The aim of the present study was to identify additional as well as cooperative genetic events in T-ALL.

**Methods:**

A population-based pediatric T-ALL series comprising 47 cases was investigated by SNP array and deep sequencing analyses of 75 genes, in order to ascertain pathogenetically pertinent aberrations and to identify cooperative events.

**Results:**

The majority (92%) of cases harbored copy number aberrations/uniparental isodisomies (UPIDs), with a median of three changes (range 0–11) per case. The genes recurrently deleted comprised *CDKN2A*, *CDKN2B*, *LEF1*, *PTEN*, *RBI*, and *STIL*. No case had a whole chromosome UPID; in fact, literature data show that this is a rare phenomenon in T-ALL. However, segmental UPIDs (sUPIDs) were seen in 42% of our cases, with most being sUPID9p that always were associated with homozygous *CDKN2A* deletions, with a heterozygous deletion occurring prior to the sUPID9p in all instances. Among the 75 genes sequenced, 14 (19%) were mutated in 28 (72%) of 39 analyzed cases. The genes targeted are involved in signaling transduction, epigenetic regulation, and transcription. In some cases, *NOTCH1* mutations were seen in minor subclones and lost at relapse; thus, such mutations can be secondary events.

**Conclusions:**

Deep sequencing and SNP array analyses of T-ALL revealed lack of wUPIDs, a high proportion of sUPID9p targeting *CDKN2A*, *NOTCH1* mutations in subclones, and recurrent mutations of genes involved in signaling transduction, epigenetic regulation, and transcription.

**Electronic supplementary material:**

The online version of this article (doi:10.1186/s13045-015-0138-0) contains supplementary material, which is available to authorized users.

## Background

T-cell acute lymphoblastic leukemia (T-ALL) arises through a stepwise acquisition of genetic aberrations that occasionally are important for proper diagnosis and prognostication as well as for therapeutic decisions [1]. Cytogenetically, approximately half of T-ALL cases display clonal chromosome aberrations, with the cytogenetic and oncogenic hallmark of T-ALL being the presence of translocations leading to illegitimate rearrangements of the T-cell receptor (TCR) loci (mainly *TRA/TRD* at 14q11 and *TRB* at 7q34). Genes targeted, and hence deregulated, by TCR rearrangements most often code for transcription factors or proteins involved in transcriptional complexes, such as the *HOXA* (7p15.2), *LMO1* (11p15.4), *LMO2* (11p13), *TAL1* (1p33), *TLX1* (10q24.31), and *TLX3* (5q35.1) genes [2]. However, additional abnormalities are required for overt leukemia. Typical examples include *NOTCH1* (9q34.3) and *FBXW7* (4q31.3) mutations and *CDKN2A* (9p21.3) deletions, which are present in the vast majority of T-ALL cases [3,4]. In addition, recent array and sequence analyses have implicated deletions and/or mutations in numerous other genes, with some of the most prevalent being *ETV6* (12p13.2), *EZH2* (7q36.1), *IL7R* (5p13.2), *LEF1* (4q25), *NRAS* (1p13.2), *PHF6* (Xq26.2), *PTEN* (10q23.31), *RUNX1* (21q22.12), and *WT1* (11p13) [5]. Here, we have used single nucleotide polymorphism (SNP) array and deep sequencing analyses of 75 selected candidate genes to characterize genetic aberrations in a consecutive series of pediatric T-ALL.

## Results

### SNP array findings

**Figure 1:**
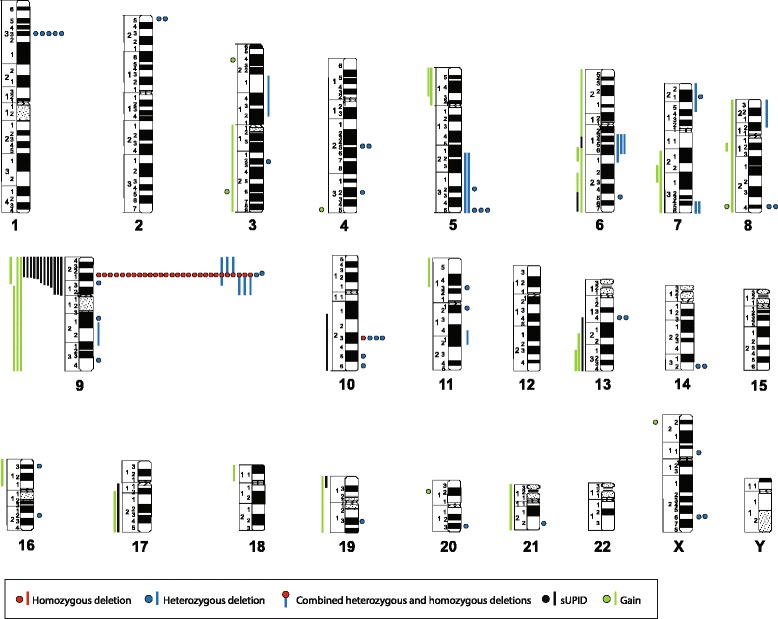
Ideogram displaying all copy number alterations (trisomies, deletions, and gains) and sUPIDs identified by SNP array analysis of 40 diagnostic T-ALL samples. Dots and bars represents aberrations <10 and ≥10 Mb, respectively. Homozygous deletions involving 9p21.3 were categorized according to the underlying mechanism: two small (<10 Mb) deletions were graphically depicted as a red dot; two heterozygous deletions, one small <10 Mb and one large ≥10 Mb, which together results in a small homozygous deletion were shown as a red dot and a blue bar combined; and sUPID9p and a homozygous deletion were indicated as a red dot and a black bar. Homozygous deletions are placed before heterozygous deletions to the right of the chromosome, and sUPIDs are placed before gains to the left. The aberration type is depicted according to size, with the largest first.

All aberrations found by SNP array analysis are listed in Additional file 1: Table S1 (using cytogenetic nomenclature) and Additional file 2: Table S2 (with detailed positions derived from the GRCh37 genome build), and are summarized graphically in Figure 1. Forty cases could be successfully analyzed. The vast majority (37/40; 92%) harbored copy number abnormalities (CNAs), i.e., trisomies, deletions, or gains, and/or segmental uniparental isodisomies (sUPIDs). No monosomies or whole chromosome UPIDs (wUPIDs) were detected. In 37 of the investigated cases, karyotypic data were also available. The SNP array analyses yielded novel information in 35 (95%) of these. In addition, the SNP arrays could better define the breakpoints and/or clarify ambiguous unbalanced changes detected by G-banding analysis in 13 (35%) cases (Additional file 1: Table S1). All abnormalities seen by chromosome banding analysis leading to net gain or loss of genetic material were confirmed by SNP array analysis.

The analyses revealed 128 CNAs, of which 88 (69%) were <10 Mb and 40 (31%) were ≥10 Mb. The median number of CNAs per case was 3 (mean 3.3, range 0–10). Deletions were more common (100/128; 78%) than gains (28/128; 22%); the latter comprised trisomies (*n* = 6) and duplications (*n* = 22). In addition, 18 sUPIDs were found, all of which ≥10 Mb. Thus, a total of 146 abnormalities (CNAs and UPDs combined) were identified, with a median number of 3 (range 0–11) aberrations per case (Figure 1 and Additional file 2: Table S2).

### Large (≥10 Mb) genomic imbalances

The only trisomies identified involved chromosomes 6, 8, 9, 19, and 21 (Figure 1). The smallest regions of overlap of recurrent large duplications were 5p13.1–pter, 7q22.1–31.2, and 13q31.1–qter, whereas the smallest regions of overlap of recurrent large deletions were 5q21.3–qter, 6q14–16, 7q34–qter (within the *TRB* locus), 9p11–21.3, and 9p21.2–pter (Figure 1 and Additional file 1: Table S1).

### Small (<10 Mb) genomic imbalances

Six genes were recurrently targeted by deletions: 1) *CDKN2A* in 29/40 (72%) cases, of which 28/29 (97%) were homozygous deletions (either two separate deletions or one deletion in combination with sUPID9p); 2) *CDKN2B* (9p21.3) in 25/40 (62%), all of which also involved *CDKN2A*; 3) *STIL* (1p33) in 5/40 (12%), all of which were verified by interphase fluorescence *in situ* hybridization (FISH) analysis using the *SIL-TAL1* sub-deletion signal FISH probe (Dako, Glostrup, Denmark); 4) *PTEN* in 3/40 (8%); 5) *LEF1* in 2/40 (5%); and 6) *RB1* (13q14.2) in 2/40 (5%). Deletions of *PHF6* and upstream elements of *LMO2* were detected in one case each (Figure 1). In four cases (#31, 34, 36, and 37), no deletions involving the TCR genes, representing somatic recombination, were detected; however, the finding of other SNP-identified aberrations and/or high levels of mutated alleles, as ascertained by deep sequencing, in these cases proved the presence of leukemic cells in the investigated samples (Additional file 1: Table S1).

### sUPIDs

Of the 18 sUPIDs identified in 17 (42%) cases, only 9p was recurrently targeted (12 cases), including *CDKN2A* and *CDKN2B* in all instances with the minimal overlap spanning 9p21.3–pter. All but one of the sUPIDs were terminal (Figure 1, Additional file 1: Table S1, and Additional file 2: Table S2). All sUPID9p were associated with homozygous *CDKN2A* deletions; *CDKN2B* was homozygously deleted in eight (67%) of the 12 cases.

### Gene mutations

All 75 genes targeted in the 39 cases analyzed could be successfully deep sequenced (2.1–3.2 million reads/sample). The mean target coverage varied between 268 times and 465 times per sample, with 98.3%–99.4% of the targets having at least 10 times coverage. Conversely, targets with no coverage ranged from 0.2% to 0.5% per sample.

A total of 46 mutations were detected among the 39 investigated cases at the time of diagnosis; the median was 1 mutation per sample (range 0–4) (Additional file 1: Table S1 and Additional file 3: Table S3). Mutations were identified in 28 (72%) of the samples. All mutations were heterozygous, except for one case with a hemizygous mutation of the *PHF6* gene at Xq26.2. Of the 46 mutations observed, 35 (76%) were missense, six (13%) indels, and five (11%) were nonsense.

Details on all mutations are given in Additional file 3: Table S3. Of the 75 genes sequenced, 14 (19%) were shown to be mutated at least once: *BCL11B* at 14q32.2 (*n* = 1), *CREBBP* at 16p13.3 (*n* = 2), *DNMT3A* at 2p23.3 (*n* = 1), *EZH2* at 7q36.1 (*n* = 1), *FBXW7* at 4q31.3 (*n* = 12; one case harbored two different mutations), *JAK1* at 1p31.3 (*n* = 1), *JAK3* at 19p13.11 (*n* = 1), *NOTCH1* at 9q34.3 (*n* = 19; two cases carried two and three different mutations, respectively), *NRAS* at 1p13.2 (*n* = 2), *PHF6* at Xq26.2 (*n* = 1), *PIK3CA* at 3q26.32 (*n* = 1), *PTEN* at 10q23.31 (*n* = 2), *SETD2* at 3p21.31 (*n* = 1), and *TCF3* at 19p13.3 (*n* = 1). Of the 19 *NOTCH1* mutations, 17 (89%) occurred in exons 26–27 (HD domain) and two (11%) in exon 34 (PEST domain). Identical mutations were found in three genes: *FBXW7* (c.1393C > T in four cases and c.1513C > T in three cases), *NOTCH1* (c.4793G > C in two cases and c.5033 T > C in three cases), and *NRAS* (c.34G > A in two cases).

### Genetic comparisons between diagnostic and relapse samples

**Figure 2:**
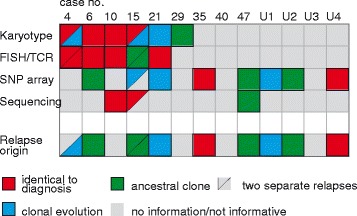
Summary of the genetic features and the genomic relationships in the 13 paired diagnostic/relapse samples.

Nine of the 47 patients relapsed, and samples from six of these were analyzed cytogenetically at both diagnosis and relapse (Figure 2 and Additional file 4: Table S4). At relapse, three cases (#4, 15, and 21) had additional aberrations. In one case (#29), the diagnostic aberration was not seen at relapse, whereas two (#6 and 10) displayed identical changes. FISH data on TCR rearrangements were available for five of the paired samples. In four of these, the FISH findings did not differ between diagnosis and relapse. In one case (#15), the diagnostic sample was positive for a *TRA*/*D* rearrangement, whereas the relapses were negative.

SNP array analyses were performed on 11 paired samples, comprising seven (#4, 6, 10, 15, 21, 35, and 47; Additional file 4: Table S4) in-house and four (#U1–U4) external cases, with informative results being obtained in eight. In two cases (#35 and U4), the aberrations at diagnosis and relapse were identical. In three cases (#15, 21, and U1), all diagnostic abnormalities were present at relapse together with additional changes (clonal evolution). In three cases (#6, 47, and U2), the diagnostic and relapse samples not only shared some aberrations but also harbored distinct aberrations (evolution from a preleukemic/ancestral clone). A total of 13 aberrations were seen only at relapse; none of these was recurrent. Targeted sequencing analyses were performed on three paired samples (cases 10, 15, and 47). In two of these (#10 and 15), the results were concordant between diagnosis and relapse. In contrast, case 47 harbored a *NOTCH1* mutation at diagnosis with a variant allele frequency (VAF) of 0.14 (Additional file 3: Table S3); this mutation was not detected at relapse.

### Survival analyses

The probabilities of relapse-free survival (pRFS), event-free survival (pEFS), and overall survival (pOS) did not differ with regard to age, white blood cell (WBC) counts, and presence/absence of TCR translocations, *STIL* deletions, *NOTCH1* and/or *FBXW7* mutations, or mutations/deletions of genes involved in epigenetic regulation or signaling transduction. The pEFS and pOS (but not pRFS) were significantly lower in girls than in boys; however, this analysis was based on few cases (Additional file 5: Table S5).

The presence of *CDKN2A* deletions was significantly associated with a high WBC count (median 209 × 10^9^/l vs. 62 × 10^9^/l; *P* = 0.013). The pEFS and pRFS did not differ in relation to *CDKN2A* status, whereas pOS was significantly lower (*P* = 0.04) for cases with *CDKN2A* deletions (Additional file 5: Table S5). Among the 29 cases with loss of *CDKN2A*, there were no significant differences between those with (*n* = 12) and without sUPID9p (*n* = 17) as regards age (median 10.9 years vs*.* 7.0 years; *P* = 0.18), gender (*P* = 0.7), or WBC count (median 242 × 10^9^/l vs. 187 × 10^9^/l; *P* = 0.25).

## Discussion

**Figure 3:**
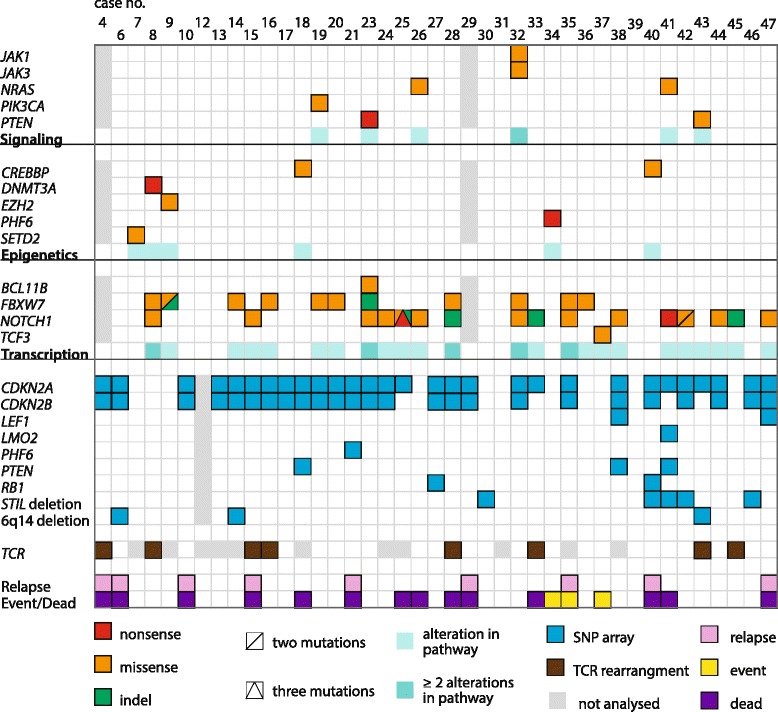
Overview of all mutations, focal deletions, 6q deletions, and TCR rearrangements identified by large-scale sequencing, SNP array, and FISH analyses of the pediatric T-ALL cohort.

The rationale for the present study, representing a truly population-based pediatric T-ALL series comprising all 47 cases diagnosed in our catchment area between 1983 and 2011, was to ascertain whether a combination of SNP array and large-scale gene mutation analyses could add pathogenetically pertinent data, in particular as regards cooperative events. Taking the findings from all these investigatory modalities into account, it is apparent that T-ALL is characterized by multiple genetic changes (Figure 3), in line with a multistep leukemogenic process [5].

Previous studies using bacterial artificial chromosome or oligonucleotide arrays have identified recurrent deletions of several genes in T-ALL, such as *BCL11B* (14q32.2), *EZH2* (7q36.1), *LEF1* (4q25), *NF1* (17q11.2), *PHF6* (Xq26.2), *PTEN* (10q23.31), and *PTPN2* (18p11.21) [6-12]. Although such investigations have yielded pathogenetically important information, the drawback of the above-mentioned techniques is that they do not provide data on UPIDs. However, surprisingly few SNP array studies of pediatric T-ALL have been reported [5,13-18], and they have generally not discussed the frequency and distribution of UPIDs. Apart from detecting a median of three CNAs, mainly deletions, per case, similar to other studies [13,17], and identifying microdeletions of *CDKN2A*, *CDKN2B*, *LEF1*, *PHF6*, *PTEN*, *RB1*, and *STIL*, we also observed a total of 18 sUPIDs targeting 6q, 9p, 10q, 13q, 17q, and 19p (Figure 1 and Additional file 1: Table S1). Interestingly, no wUPIDs were detected. In fact, when reviewing previous SNP array studies of pediatric T-ALL with data on UPIDs [13,16,17], only one out of a total of 84 cases had a wUPID at diagnosis. When adding the present 40 cases, this would translate into a frequency of 0.8% (1/124) of wUPIDs in T-ALL. The reason for the apparent lack of wUPIDs in T-ALL is unclear, but may simply be due to the fact that the vast majority of T-ALLs are pseudodiploid. Indeed, wUPIDs appear to be particularly common in aneuploid malignancies, such as hyperhaploid inflammatory leiomyosarcoma [19] and near haploid/low hypodiploid B-cell precursor ALL [20] with chromosome doubling as well as in high hyperdiploid BCP ALL [21,22]. In these cases, the wUPIDs in most instances reflect the underlying mechanism of formation.

Except for sUPID9p, the other sUPIDs, involving 6q, 10q, 13q, 17q, and 19p, were non-recurrent. However, sUPIDs affecting 10q and 17q, overlapping the sUPIDs identified in our study, have been reported in T-ALL [13,16]. The prior case with sUPID10q displayed a focal homozygous *PTEN* (10q23.31) deletion [13], suggesting that this could be the pathogenetically important outcome. In our case (#10; Additional file 1: Table S1), *PTEN* was neither deleted nor mutated. As regards sUPID17q, the gene(s) possibly involved remains to be identified. In contrast, the target of sUPID9p, detected in 30% of the present T-ALL cases, is clearly *CDKN2A* at 9p21.3. This gene was deleted in 72% of all cases, a frequency similar to previous studies [4,23,24]. In 86% of these, the neighboring *CDKN2B* gene was also deleted. Interestingly, *CDKN2A* was homozygously deleted in all 12 cases with sUPID9p, whereas *CDKN2B* was unaffected in 33% of these cases. Notably, all homozygous *CDKN2A* deletions associated with sUPID9p had identical breakpoints, strongly suggesting that a heterozygous deletion occurred prior to the sUPID.

In one of the investigated cases (U1), the SNP array analysis revealed a complex rearrangement of 6q, with a total of 28 breakpoints between 6q14 and 6qter and with copy number changes oscillating between one and two copies (Additional file 6: Figure SI), representing chromothripsis [25]. This phenomenon has been described in numerous tumor types [26], including a few cases of early T-cell precursor ALL [5]. Notably, the 6q abnormality in case U1 also harbored a larger proximal deletion, involving, among others, the *CASP8AP2* gene at 6q15. This gene has been implicated as a prognostic marker in T-ALL [7,27] and was deleted in all our T-ALL cases with del(6q).

Most previous mutation analyses of T-ALL have focused on only one or a few candidate genes, for example *ETV6* (12p13.2), *FBXW7* (4q31.3), *FLT3* (13q12.2), *IL7R* (5p13.2), *IRS4* (Xq22.3), *JAK1* (1p31.3), *NOTCH1* (9q34.3), *NRAS* (1p13.2), and *TP53* (17p13.1). Although such an approach has been fruitful, it does not provide data on cooperative mutations in leukemogenesis [3,28-34]. More recently, studies applying whole genome or exome sequencing have been forthcoming, reporting an average of approximately ten protein altering mutations per pediatric T-ALL case [5,35,36]. In this study, we established a gene panel comprising 75 genes previously reported to be mutated in T-ALL or shown to encode factors of importance in hematopoiesis. Using this approach, we obtained a mean sequencing depth of between 268 times and 465 times per sample, enabling detection of very small subclones.

Of the 39 samples sequenced, 28 (72%) had one or several mutations, and of the 75 genes, 14 (19%) were found to be mutated at least once. The genes targeted can broadly be categorized into one of the three groups: signaling transduction (*JAK1*, *JAK3*, *NRAS*, *PI3KCA*, and *PTEN* mutations were seen in 15% of cases), epigenetic regulation (*CREBBP*, *DNMT3A*, *EZH2*, *PHF6*, and *SETD2*; 15%), and transcription (*BCL11B*, *FBXW7*, *NOTCH1*, and *TCF3*; 59%) (Figure 3). More than 75% of the mutations were present in major clones, as ascertained by VAFs above 0.25 (Additional file 3: Table S3). Because mutations may be misinterpreted as subclonal due to admixture of non-neoplastic cells, we only considered mutations with a VAF <0.25 to represent minor subclones if additional data showed a high proportion of malignant cells, i.e., if co-occurring mutations or CNAs/sUPIDs were detected at higher frequencies. Using these criteria, subclones with *FBXW7*, *NOTCH1*, and/or *PTEN* were identified in six cases (Additional file 3: Table S3). *NOTCH1* has been suggested to act as an initiating event in T-ALL [37,38]. However, the present findings, together with previous studies reporting low-level mutation frequencies of *NOTCH1* and relapses of *NOTCH1*-positive T-ALL being negative for the mutation, as also seen in our case 47 (Additional file 4: Table S4) [39,40], show that *NOTCH1* mutations also can be secondary events.

By combining the cytogenetic, FISH, SNP array, and mutation results in paired diagnostic and relapse samples, three different evolution patterns emerged: i) identical clones, ii) clonal evolution, and iii) ancestral clones (Figure 2 and Additional file 4: Table S4), in line with prior studies [23,36,40]. In no instance did we have any evidence for completely distinct clones at relapse. Thus, none of the relapses could be considered a “second” leukemia, as has been suggested in a few instances of T-ALL relapses [16]. However, the latter study focused on late relapses, i.e., those occurring more than 2.5 years after diagnosis, and most of our cases were early relapses. Quite few additional genetic changes were present at relapse (Additional file 4: Table S4), confirming that genomic instability is not a major feature of T-ALL [15].

## Conclusion

The salient findings in the present study were the lack of wUPIDs, a high proportion of sUPID9p targeting *CDKN2A*, *NOTCH1* mutations in subclones, and recurrent mutations of genes involved in signaling transduction, epigenetic regulation, and transcription in T-ALL.

## Methods

### Patients and cytogenetic/FISH analyses

Between 1983 and 2011, 47 children/adolescents (<18 years) were diagnosed with T-ALL in southern Sweden (Departments of Pediatric Hematology and Oncology, Lund and Linköping University Hospitals; Additional file 1: Table S1). The T-ALL diagnosis was based on immunophenotypic feature [41]. The male/female ratio was 4.2, median age 9.1 years (range 0.7–17.2 years), and the median WBC count was 92 × 10^9^/l (range 1.4–768 × 10^9^/l). Mediastinal and central nervous system involvement was seen in 62% and 13% of informative cases, respectively (Additional file 7: Table S6). The pRFS, pEFS, and pOS at both 5 and 10 years were 0.815 (0.06), 0.604 (SE 0.07), and 0.665 (SE 0.07), respectively. The basic clinical features did not differ significantly (chi-square test) from those observed in a population-based cohort of 285 pediatric Nordic T-ALL patients previously reported by us [42]. Samples from 44 (94%) of the patients were sent for cytogenetic analysis to the Department of Clinical Genetics, Lund University Hospital, Sweden. The median number of analyzed metaphases per case was 17 (range 0–49). All abnormal karyotypes have been centrally reviewed annually since 1996 by the Swedish Childhood Leukemia Cytogenetics Group, and five different treatment protocols were used during this time period by the Nordic Society for Pediatric Hematology and Oncology (NOPHO): NOPHO ALL-1981, −1986, −1992, −2000, and −2008 [43,44]. The study was approved by the Regional Ethical Review Board at Lund University, and informed consent was obtained according to the Declaration of Helsinki.

In the present study, interphase FISH analyses using the *TRA/TRD* break apart FISH probe (Abbott, Stockholm, Sweden) and the *TRB* split signal FISH probe (Dako) were carried out on 31 diagnostic samples from which cells in fixative were available. Cutoff values (median +3 SD) for *TRA/TRD* and *TRB* rearrangements, based on analyses of five normal bone marrow (BM) samples, were 4.9% and 1.1%, respectively. TCR rearrangements, as ascertained by interphase FISH analysis, were identified in eight (26%) of the 31 cases investigated; five involved *TRA/TRD* (#8, 15, 16, 28, and 33) and three *TRB* (#4, 43, and 45). Six of these had been detected by G-banding analysis (#8, 16, 28, 33, 43, and 45), whereas two cases (4 and 15) had seemingly normal karyotypes (Additional file 1: Table S1). The frequency of FISH-identified TCR translocations (26%), with some of them being cytogenetically cryptic, is in line with prior reports [45,46].

### SNP array analysis—diagnostic samples

DNA was available from 40 of the 44 samples and was extracted from BM (*n* = 33) or peripheral blood (*n* = 7); in 27/40 (68%) cases, a remission sample could be included as a control. The HumanOmni1-Quad BeadChip, containing >1 million markers with a median marker spacing of 1.5 kb (Illumina, San Diego, CA, USA), was used. The analyses were performed according to the manufacturer’s instructions, and data analysis was done using the GenomeStudio software 2011.1 (Illumina), extracting probe positions from the GRCh37 genome build (http://www.ensembl.org/Homo_sapiens/Info/Index). Imbalances seen in remission samples or overlapping with copy number polymorphisms listed in the Database of Genomic Variants (http://projects.tcag.ca/variation/) were excluded from further analysis, and so were deletions involving the TCR and immunoglobulin loci because they most likely represent somatic rearrangements clonotypic for the malignant lymphoid cells rather than oncogenic events [47].

### SNP array analysis—paired diagnostic/relapse samples

Of the 47 patients with T-ALL, nine relapsed (Additional file 7: Table S6). SNP array analyses could be performed on diagnostic as well as on relapse samples from seven of these (Additional file 1: Table S1 and Additional file 4: Table S4). These samples were analyzed using the Illumina platform described above. In addition, four paired diagnostic/relapse T-ALL (cases U1–U4) samples from the Department of Pediatric Hematology and Oncology, Norrland University Hospital, Umeå, Sweden, were available for analysis; these were investigated using the Illumina Humancnv370-Duo BeadChip containing >370,000 markers, with a median marker spacing of 4.9 kb (Illumina). The analyses were performed as above with the exception that the probe positions were extracted from the NCBI36.1 genome build.

### Targeted deep sequencing of 75 genes

A sufficient amount of DNA for large-scale sequencing was available from 39 diagnostic and three relapse samples (Additional file 1: Table S1). The DNA quality was evaluated using the NanoDrop 1000 Spectrophotometer (Thermo Scientific, Wilmington, DE, USA) and by visual inspection of gel images of total genomic DNA. Seventy-five genes, comprising genes previously reported to be mutated in T-ALL or to participate in various cellular processes in T-cells, such as signaling transduction, epigenetic regulation, transcription, cell cycle control, cell-cell interaction, and DNA repair (Additional file 8: Table S7), were targeted for capture and deep sequencing. Using the eArray system (Agilent Technologies, Santa Clara, CA, USA), a SureSelect capture library was designed to include all exons and flanking intronic sequences. In addition, 5′UTR and 3′UTR were added to the selected regions. A total of 15 343 baits, which covered 95% of the target bases, corresponded to a final capture size of 0.5 Mb. After DNA fragmentation, which yielded fragments of a median size of 251 bp, sequencing libraries were generated using the SureSelect post capture protocol for Illumina paired-end sequencing (Agilent). The samples were sequenced on an Illumina HiSeq 2000 platform using 100-bp paired-end reads. The sequences were aligned to the human genome reference hg 19 (http://hgdownload.cse.ucsc.edu/downloads.html#human), with the short read alignment program Burrows-Wheeler Alignment tool [48]. Library preparation, sequencing, and alignment were performed by Science for Life Laboratory (SciLifeLab, Solna, Sweden). After alignment, each sample was evaluated by performing filtering and variant calling using the Genespring GX software (Agilent), including removal of duplicates, a confident score cutoff of 10, base quality of 30, and ignorance of reference locations under 5/1.

Only variants classified as non-synonymous and not present as germline SNPs in the dbSNP database were retained. Of the remaining variants, those fulfilling the following criteria were considered pathogenetically relevant: i) known cancer-associated mutations listed in the Ensembl genome browser (http://www.ensembl.org/index.html) and ii) novel variants that could be confirmed by Sanger sequencing of diagnostic samples and that were not present in remission samples. Sanger dideoxy terminator sequencing was performed with primers designed with Primer3web (http://bioinfo.ut.ee/primer3/); primer sequences are available upon request. PCR was done using the BigDye® Terminator v1.1 Cycle Sequencing Kit (Applied Biosystems, Foster City, CA, USA), and the sequencing products were resolved on the 3130 Genetic Analyzer (Applied Biosystems).

### Statistical analyses

The IBM SPSS Statistics for Windows, Version 22 (Armonk, IBM Corp, NY, USA) was used for all statistical analyses. The significance limit for two-sided *P* values was set to <0.05. The Mann–Whitney *U* test was used to investigate possible correlations between *CDKN2A* deletions and age and WBC count. Fisher exact probability and Mann–Whitney *U* tests were used to compare the distribution of gender, age, and WBC counts between *CDKN2A* deleted cases with and without sUPID9p. The pRFS, pEFS, and pOS were calculated using the Kaplan-Meier method, and subgroups were compared using the log-rank test. The following parameters were compared: clinical features (age, gender, and WBC count), TCR translocations, SNP array findings, i.e., number of aberrations (CNAs and sUPIDs combined), and presence/absence of *CDKN2A* and *STIL* deletions, *NOTCH1* and/or *FBXW7* mutations, and mutations/deletions of genes encoding proteins involved in epigenetic regulation or signaling transduction. In the analysis of pEFS, events comprised induction failure, relapse, second malignant neoplasm, and death in complete remission 1. In the OS analyses, death of any cause was the endpoint. Patients in continuous complete remission 1 were followed up between 0 and 304 months (median 66 months). The date of the last follow-up was January 10, 2012.

## Additional files

Additional file 1: Table S1. Genetic features of the 47 T-ALL cases diagnosed in southern Sweden 1983–2011. Additional file 2: Table S2. Abnormalities detected by SNP array analysis. Additional file 3: Table S3. Detailed information on the 46 identified mutations. Additional file 4: Table S4. Genetic features of diagnostic and relapse samples. Additional file 5: Table S5. Survival in relation to clinical and genetic features. Additional file 6: Figure S1. SNP array analysis results for 6q on case U1. The B allele frequency and log R ratio oscillates between heterozygous/homozygous states and two/one copies, respectively, representing chromothripsis. Additional file 7: Table S6. Clinical and immunophenotypic features of the 47 T-ALL patients diagnosed in southern Sweden 1983–2011. Additional file 8: Table S7. The 75 genes sequenced and their chromosomal positions.
