# Supplementary material for: Deep sequencing and SNP array analyses of pediatric T-cell acute lymphoblastic leukemia reveal NOTCH1 mutations in minor subclones and a high incidence of uniparental isodisomies affecting CDKN2A
Source: J Hematol Oncol. 2015 Apr 24;8:42. doi: 10.1186/s13045-015-0138-0 (PMC4412034; doi:10.1186/s13045-015-0138-0)
Supplement: Additional file 1: Table S1. — Genetic features of the 47 T-ALL cases diagnosed in southern Sweden 1983–2011. [file 13045_2015_138_MOESM1_ESM.docx]

**Additional file 1: Table S1.** Genetic features of the 47 T-ALL cases diagnosed in southern Sweden 1983-2011

| Case  No. | Karyotype/  Imbalances/UPIDs identified by SNP array analysis | TCR  (FISH) | Mutated genes^a^ |
| --- | --- | --- | --- |
| 1 | 45,XY,add(5)(p15),−8^b^  No DNA for SNP array analysis | NA | NA |
| 2 | NA  No DNA for SNP array analysis | NA | NA |
| 3 | NA  No DNA for SNP array analysis | NA | NA |
| 4D | 46,XY^b^  del(9)(p21.3p21.3)x2,UPID(9)(p13.3pter) | *TRB* | NA |
| 4R1 | 46,XY  No changes identified by SNP array^c^ | *TRB* | NA |
| 4R2 | 46,XY,t(7;11)(q35;p13)/46,idem,del(6)(q21q25),  add(17)(p13)^b^  No DNA for SNP array analysis | *TRB* | NA |
| 5 | 46,XY,del(9)(p13)^b^  No DNA for SNP array analysis | No | NA |
| 6D | 46,XY,del(6)(q13q21)^b^  del(6)(q13q16.3),del(9)(p21.3p21.3)x2,UPID(9)  (p21.1pter) | No | No mutations |
| 6R | 46,XY,del(6)(q13q21)  del(2)(p16.3p16.3),del(6)(q14.1q22.31),del(9)  (p21.3p21.3)x2,UPID(9)(p21.1pter) | No | NA |
| 7 | 46,XY  del(5)(q21.3qter),dup(6)(q22.31q24.2),UPID(6)  (q24.2qter),dup(8)(q11.23q12.3),dup(13)  (q21.32qter),del(20)(q13.12q13.12) | NA | *SETD2* (p.R1625H) |
| 8 | 46,XY,t(7;14)(q36;q11-12)^b^  del(10)(q25.1q25.2) | *TRA/D* | *DNMT3A* (p.R320X), *FBXW7* (p.Y545C), *NOTCH1* (p.L1709P) |
| 9 | 46,XY,t(10;11)(p13;q21)^b^  No changes identified by SNP array | NA | *EZH2* (p.R502P), *FBXW7* (p.R441W, p.R465V) |
| 10D | 46,XY  del(9)(p21.3p21.3)x2,UPID(10)(q21.3qter) | No | No mutations |
| 10R | 46,XY  No changes identified by SNP array^c^ | No | No mutations |
| 11 | 46,XY  No DNA for SNP array analysis | NA | NA |
| 12 | 46,XY  No DNA for SNP array analysis | NA | No mutations |
| 13 | 46,XY  del(9)(p11p21.3),del(9)(p21.3p21.3) | NA | No mutations |
| 14 | 46,XX,del(9)(p12-13)^b^  del(6)(q13q16.1),del(9)(p11p21.3),del(9)  (p21.3p21.3) | NA | *FBXW7* (p.R505C) |
| 15D | 46,XY^b^  dup(8)(q24.21q24.21),del(9)(p21.3p21.3)x2 | *TRA/D* | *NOTCH1* (p.I1680N) |
| 15R1 | 46,XY  del(8)(q21.3q21.3),dup(8)(q24.21q24.21),del(9)  (p21.3p21.3)x2 | No | *NOTCH1* (p.I1680N) |
| 15R2 | 46,XY,del(6)(p12),der(17)t(6;17)(p12;p11),del(20)  (q11)^b^  No DNA for SNP array analysis | No | NA |
| 16 | 45,XY,der(8;9)(q10;q10),add(10)(p15),  t(11;14)(p13;q11)^b^  del(2)(p25.1p25.1),del(2)(p25.1p25.2),  del(8)(p12pter),del(9)(p13.2p13.3),del(9)  (p21.3p21.3),del(9)(p21.1pter),dup(17)(q11.2qter) | *TRA/D* | *FBXW7* (p.R505C) |
| 17 | Karyotypic failure  del(9)(p21.3p21.3)x2,UPID(9)(p11pter) | No | No mutations |
| 18 | 46,XY^b^  del(5)(q32q32),del(5)(q35.1q35.1),del(5)  (q35.2q35.3),del(9)(p13.1pter),del(9)(p21.3p21.3),  dup(9)(p13.1qter),del(10)(q23.31q23.31),  del(14)(q32.2q32.2) | NA | *CREBBP* (p.N705S) |
| 19 | 47,XY,+?8^b^  +8,del(9)(p21.3p21.3)x2 | No | *FBXW7* (p.R465H), *PIK3CA* (p.H1047R) |
| 20 | 46,XY^b^  del(X)(q26.3q26.3),dup(4)(q35.2q35.2),+6,UPID(6)  (q13q16.1),del(7)(p21.2p21.2),del(8)(q24.1q24.1),  +9,del(9)(p21.3p21.3)x2,+19,+21 | No | *FBXW7* (p.R465C) |
| 21D | 46,XY^b^  del(X)(q26.2q26.2),del(9)(p21.3p21.3)x2 | No | No mutations |
| 21R | 47,XY,−8,−13,?der(17)t(13;17)(q1?;p1?),+19,  +2mar^b^  del(X)(q26.2q26.2),del(8)(pterq13.1),del(9)  (p21.3p21.3)x2,del(9)(q34.2q34.3),dup(13)  (q10q12.13),UPID(13)(q12.13q14.13),del(13)  (q14.13qter),del(17)(p13.3pter),+19,del(20)  (q13.32q13.33) | No | NA |
| 22 | NA  No DNA for SNP array analysis | NA | NA |
| 23 | 46,XY^b^  del(9)(p21.3p21.3)x2,UPID(9)(p21.1pter) | No | *BCL11B* (p.T450A), *FBXW7* (p.N401delN), *NOTCH1* (p.L1593P), *PTEN* (p.G293X) |
| 24 | 46,XX^b^  del(9)(p21.3p21.3)x2 | NA | *NOTCH1* (pV1578G) |
| 25 | 46,XY^b^  del(9)(p21.3p21.3)x2,UPID(9)(p13.2pter) | NA | *NOTCH1* (p.V1578delV, p.L1678P, p.E2460X) |
| 26 | 47,XX,+r,inc^b^  dup(X)(p22.33p22.33),dup(3)(p24.1p24.1),del(5)  (q21.3qter),del(7)(p14.1pter),del(7)(q34qter),dup(7)  (q22.1q31.2),del(9)(q34.11q34.11),dup(13)  (q31.1qter),del(19)(q13.33q13.41) | No^d^ | *NOTCH1* (p.V1721E), *NRAS* (p.G12S) |
| 27 | 46,XY^b^  del(9)(p21.3p21.3)x2,del(10)(q26.2q26.2),del(13)  (q14.2q14.2),del(21)(q22.3q22.3) | No | No mutations |
| 28 | 46,Y,t(X;14)(p11;q11)^b^  del(4)(q32.3q32.3),del(5)(q35.1q35.1),del(9)  (p21.3p21.3)x2,del(14)(q32.2q32.2),del(16)  (q22.1q22.1) | *TRA/D* | *FBXW7* (p.R465C), *NOTCH1* (p.F1606>LD) |
| 29D | 47,XY,+9^b^  dup(3)(q26.32q26.32),del(8)(q24.1q24.1),+9,del(9)  (p21.3p21.3)x3 | No | NA |
| 29R | 46,XY  No DNA for SNP array analysis | NA | NA |
| 30 | 46,XY,add(3)(p13)  del(1)(p33p33),del(3)(p12.2p21.31),del(16)  (p13.3p13.3) | No | No mutations |
| 31 | 46,XY  dup(3)(q11qter),dup(5)(p13.1pter),dup(9)  (p13.2pter) | NA | No mutations |
| 32 | 46,XY,add(9)(p13)^b^  del(X)(p11.23p11.23),del(9)(p21.3p21.3)x2,  UPID(9)(p11pter),dup(18)(p11pter) | No | *JAK1* (p.R724H), *JAK3* (p.M511I), *FBXW7* (p.R505C), *NOTCH1* (p.L1547P) |
| 33 | 47,XX,t(12;14)(p13;q11),+mar^b^  del(9)(p21.3p21.3)x2,UPID(9)(p21.1pter),dup(16)  (p11pter) | *TRA/D* | *NOTCH1* (p.P1582delP) |
| 34 | 46,XY,?t(11;12)(q13;q23),?del(21)(q22)^b^  del(6)(q25.3q25.3),del(11)(p12.1p12.2) | No | *PHF6* (p.R319X) |
| 35D | Karyotypic failure  del(9)(p21.3p21.3)x2 | NA | *FBXW7* (p.R465C), *NOTCH1* (p.L1678P) |
| 35R | 46,XX  del(9)(p21.3p21.3)x2 | NA | NA |
| 36 | 46,XY^b^  No changes identified by SNP array | No | *FBXW7* (p.R465C) |
| 37 | 46,XY  UPID(13)(q14.11qter) | No | *TCF3* (p.A8V) |
| 38 | 46,XY,del(9)(p21)^b^  del(4)(q25q25),del(7)(q34qter),del(9)(p11p21.3),  del(9)(p21.3p21.3),del(10)(q23.31q23.31),  dup(11)(p13pter) | NA | *NOTCH1* (p.L1596H) |
| 39 | Karyotypic failure  No changes identified by SNP array | No | No mutations |
| 40D | 46,XX  del(1)(p33p33),dup(6)(q16.1q21),del(9)  (p21.3p21.3)x2,UPID(9)(p13.3pter),del(13)  (q14.2q14.2),dup(20)(p12.1p12.2) | No | *CREBBP* (p.Q2208H) |
| 40R | NA  No DNA for SNP array analysis | NA | NA |
| 41 | 46,XX  del(1)(p33p33),del(9)(p21.3p21.3)x2,UPID(9)  (p21.3pter),del(10)(q23.31q23.31)x2,del(11)  (p13p13) | No | *NOTCH1* (p.S2492X), *NRAS* (p.G12S) |
| 42 | 46,XY  del(1)(p33p33),del(9)(p21.3p22.1)x2,UPID(9)  (p13.2pter),UPID(19)(p13.12pter) | No | *NOTCH1* (p.L1585P, p.R1598P) |
| 43 | 46,XY,del(6)(q?23),t(7;11)(q34;p13)  del(6)(q13q16.3),del(9)(p21.3p21.3)x2,UPID(9)  (p13.3pter) | *TRB* | *PTEN* (p.D236E) |
| 44 | 46,XY  del(3)(q22.3q23),del(9)(p21.3p21.3),del(9)  (p21.2pter),del(9)(q21.13q21.13),del(9)  (q21.2q31.2) | No | *NOTCH1* (p.L1678P) |
| 45 | 46,XX,t(6;7)(q23;q34)  del(11)(q14.1q22.1) | *TRB* | *NOTCH1* (p.V1604Q) |
| 46 | 47,XY,+mar  del(1)(p33p33),dup(5)(p11pter),dup(7)(q21.11qter),  del(9)(p21.3p21.3)x2,UPID(9)(p21.1pter) | No | No mutations |
| 47D | 46,XX  del(4)(q25q25),del(9)(p21.3p21.3),UPID(17)  (q11.2qter) | No | *NOTCH1* (p.R1598P) |
| 47R | NA  UPID(17)(q11.2qter) | NA | No mutations |

D, diagnosis; FISH, fluorescence *in situ* hybridization; NA, not analyzed; R, relapse; SNP, single nucleotide polymorphism; T-ALL, T-cell acute lymphoblastic leukemia; TCR, T-cell receptor rearrangement; UPID, uniparental isodisomy.

^a^Based on sequencing of 75 genes previously reported to be mutated in T-ALL or to participate in important T-cell associated signalling cascades (see Additional file 7: Table S7).

^b^These karyotypes have previously been reported [[1-4](#_ENREF_1)].

^c^Most likely low tumor cell count because there were no signs of TCR rearrangements or other clonal changes in the SNP array analysis.

^d^Split *TRB* FISH signal with loss of the telomeric probe.

**References**

1. Heim S, Békassy AN, Garwicz S, Heldrup J, Kristoffersson U, Mandahl N et al. Bone marrow karyotypes in 94 children with acute leukemia. Eur J Haematol. 1990;44:227-33.

2. Andreasson P, Höglund M, Békassy AN, Garwicz S, Heldrup J, Mitelman F et al. Cytogenetic and FISH studies of a single center consecutive series of 152 childhood acute lymphoblastic leukemias. Eur J Haematol. 2000;65:40-51.

3. Karrman K, Isaksson M, Paulsson K, Johansson B. The insulin receptor substrate 4 gene (*IRS4*) is mutated in paediatric T-cell acute lymphoblastic leukaemia. Br J Haematol. 2011;155:516-9.

4. Olsson L, Castor A, Behrendtz M, Biloglav A, Forestier E, Paulsson K et al. Deletions of *IKZF1* and *SPRED1* are associated with poor prognosis in a population-based series of pediatric B-cell precursor acute lymphoblastic leukemia diagnosed between 1992 and 2011. Leukemia. 2014;28:302-10.
