# Supplementary material for: Deep sequencing and SNP array analyses of pediatric T-cell acute lymphoblastic leukemia reveal NOTCH1 mutations in minor subclones and a high incidence of uniparental isodisomies affecting CDKN2A
Source: J Hematol Oncol. 2015 Apr 24;8:42. doi: 10.1186/s13045-015-0138-0 (PMC4412034; doi:10.1186/s13045-015-0138-0)
Supplement: Additional file 2: Table S2. — Abnormalities detected by SNP array analysis. [file 13045_2015_138_MOESM2_ESM.doc]

**Additional file 2: Table S2.** Abnormalities detected by SNP array analysis

| Case | Chr | Abnormality | Size^a^  (Mb) | LNS | Pos LNS | FAS | Pos FAS | LAS | Pos LAS | FNS | Pos FNS |
| --- | --- | --- | --- | --- | --- | --- | --- | --- | --- | --- | --- |
| 1 |  | NA |  |  |  |  |  |  |  |  |  |
| 2 |  | NA |  |  |  |  |  |  |  |  |  |
| 3 |  | NA |  |  |  |  |  |  |  |  |  |
| 4D | 9 | UPID | 33.8 |  |  | pter |  | rs706142 | 33796157 | rs7042499 | 34162975 |
|  | 9 | 0 copies | 0.37 | kgp22845987 | 21772518 | kgp22845987 | 21772518 | kgp5284357 | 22137765 | kgp18668814 | 22201915 |
| 4R |  | NC |  |  |  |  |  |  |  |  |  |
| 5 |  | NA |  |  |  |  |  |  |  |  |  |
| 6D | 6 | 1 copy | 34.1 | rs7745125 | 70075331 | rs3799095 | 70082863 | rs9377572 | 104180114 | rs9499708 | 104445367 |
|  | 9 | UPID | 30.5 |  |  | pter |  | rs4878432 | 30490252 | rs4242682 | 30510322 |
|  | 9 | 0 copies | 0.24 | kgp4583131 | 21798131 | kgp4583131 | 21798131 | kgp11451597 | 22040765 | kgp18541261 | 22041890 |
| 6R | 2 | 1 copy | 0.02 | rs3136245 | 48012846 | rs2020908 | 48026308 | rs2651766 | 48044772 | rs12463595 | 48098708 |
|  | 6 | 1 copy | 46.8 | rs7761239 | 78390094 | rs10943488 | 78456439 | rs781720 | 125220250 | rs781739 | 125232333 |
|  | 9 | UPID | 30.5 |  |  | pter |  | rs4878432 | 30490252 | rs4242682 | 30510322 |
|  | 9 | 0 copies | 0.24 | kgp4583131 | 21798131 | kgp4583131 | 21798131 | kgp11451597 | 22040765 | kgp18541261 | 22041890 |
| 7 | 5 | 1 copy | 74.8 | rs10070246 | 106084494 | rs1898626 | 106144139 | qter |  |  |  |
|  | 6 | 3 copies | 22.1 | rs9398707 | 123226577 | rs9375219 | 123414078 | rs12190534 | 145524534 | rs9390307 | 145677803 |
|  | 6 | UPID | 25.4 | rs9390307 | 145677803 | rs9390307 | 145677803 | qter |  |  |  |
|  | 8 | 3 copies | 10.5 | kgp664254 | 55426299 | kgp4322441 | 55478939 | rs2956318 | 65989511 | rs7842485 | 66078557 |
|  | 13 | 3 copies | 47.6 | rs9317622 | 67447544 | rs17081990 | 67476990 | qter |  |  |  |
|  | 20 | 1 copy | 0.35 | rs2018876 | 45782006 | rs2903940 | 45796660 | rs1206882 | 46147566 | rs6094752 | 46256424 |
| 8 | 10 | 1 copy | 3.69 | rs11194157 | 110311094 | rs2482595 | 110354417 | rs12411967 | 114043310 | rs12098574 | 114215769 |
| 9 |  | NC |  |  |  |  |  |  |  |  |  |
| 10D | 9 | 1 copy | 0.12 | kgp9393505 | 21895053 | kgp8516614 | 21901618 | kgp823150 | 22020493 | kgp22846646 | 22027703 |
|  | 9 | 0 copies | 0.03 | kgp18390925 | 21958279 | kgp18390925 | 21958279 | kgp18251351 | 21990546 | kgp22853822 | 21991106 |
|  | 10 | UPID | 70.4 | rs1046521 | 64914467 | rs10995495 | 65062057 | qter |  |  |  |
| 10R |  | NC |  |  |  |  |  |  |  |  |  |
| 11 |  | NA |  |  |  |  |  |  |  |  |  |
| 12 |  | NA |  |  |  |  |  |  |  |  |  |
| 13 | 9 | 1 copy | 26.6 | rs10811376 | 20617114 | rs2188229 | 20625875 | cen |  |  |  |
|  | 9 | 0 copies | 0.15 | rs13302653 | 21833046 | rs13302653 | 21833046 | rs4074785 | 21981583 | rs2811711 | 21993964 |
| 14 | 6 | 1 copy | 25.9 | cnvi0000383 | 70459788 | rs2305840 | 70500518 | rs612612 | 96444545 | rs9322615 | 96481452 |
|  | 9 | 1 copy | 25.7 | rs4978121 | 21494300 | rs9298816 | 21555283 | cen |  |  |  |
|  | 9 | 0 copies | 0.45 | kgp9163037 | 21773541 | kgp9163037 | 21773541 | kgp18650560 | 22219770 | kgp18653637 | 22227987 |
| 15D | 8 | 3 copies | 0.27 | rs997310 | 129920280 | rs987525 | 129949154 | rs13280665 | 130219805 | rs10808577 | 130248593 |
|  | 9 | 1 copy | 2.41 | rs7046563 | 20713087 | rs12375887 | 20910537 | rs10811888 | 23325084 | rs1411730 | 23338258 |
|  | 9 | 0 copies | 0.64 | rs10491569 | 21456776 | rs10491569 | 21456776 | rs10757274 | 22096055 | kgp22843039 | 22097287 |
| 15R1 | 8 | 1 copy | 0.18 | rs958401 | 87591778 | rs1441249 | 87614243 | rs17620495 | 87799007 | rs7010183 | 87801378 |
|  | 8 | 3 copies | 0.27 | rs997310 | 129920280 | rs987525 | 129949154 | rs13280665 | 130219805 | rs10808577 | 130248593 |
|  | 9 | 1 copy | 2.41 | rs7046563 | 20713087 | rs12375887 | 20910537 | rs10811888 | 23325084 | rs1411730 | 23338258 |
|  | 9 | 0 copies | 0.64 | rs10491569 | 21456776 | rs10491569 | 21456776 | rs10757274 | 22096055 | kgp22843039 | 22097287 |
| 16 | 2 | 1 copy | 2.36 | rs7600748 | 6794618 | cnvi10107843 | 6805358 | rs1473101 | 9165288 | rs6431973 | 9185559 |
|  | 2 | 1 copy | 0.72 | rs6727702 | 10175442 | rs6432057 | 10210244 | rs4799 | 10933249 | rs1734343 | 10937037 |
|  | 8 | 1 copy | 32.4 |  |  | pter |  | rs2466062 | 32443090 | rs2976532 | 32594251 |
|  | 9 | 1 copy | 31.6 |  |  | pter |  | rs12337314 | 31555977 | rs1412341 | 31619571 |
|  | 9 | 0 copies | 0.56 | kgp7319441 | 21760254 | kgp7319441 | 21760254 | kgp1167283 | 22323162 | kgp5064075 | 22317743 |
|  | 9 | 1 copy | 4.51 | rs10971720 | 33827153 | rs2050789 | 33880690 | rs4646770 | 38393171 | rs5013198 | 38489712 |
|  | 17 | 3 copies | 55.0 | rs10459953 | 26127518 | rs9905137 | 26167406 | qter |  |  |  |
| 17 | 9 | UPID | 47.3 |  |  | pter |  | cen |  |  |  |
|  | 9 | 0 copies | 1.14 | rs1330317 | 21256062 | rs1330317 | 21256062 | kgp5491039 | 22391702 | rs7024019 | 22392359 |
| 18 | 5 | 1 copy | 0.37 | rs9325009 | 145804417 | rs765913 | 145918244 | rs471172 | 146286747 | rs470521 | 146554431 |
|  | 5 | 1 copy | 1.05 | rs153750 | 171181237 | rs33857 | 171193790 | rs7715908 | 172243695 | rs931609 | 172300401 |
|  | 5 | 1 copy | 1.32 | rs1592792 | 175465537 | rs13181538 | 175797836 | rs10060053 | 177120697 | rs11738486 | 177140874 |
|  | 9 | 1 copy | 38.8 |  |  | pter |  | rs10116618 | 38769003 | cnvi0147832 | 38809165 |
|  | 9 | 0 copies | 0.95 | kgp22850718 | 21915503 | kgp22850718 | 21915503 | kgp18321656 | 22010076 | kgp18321656 | 22010076 |
|  | 9 | 3 copies | 102 | cnvi0147832 | 38809165 | cnvi0147832 | 38809165 | qter |  |  |  |
|  | 10 | 1 copy | 0.12 | rs2038554 | 89534249 | rs12241293 | 89566989 | rs17107001 | 89686509 | rs7078674 | 89697353 |
|  | 14 | 1 copy | 0.33 | rs1023173 | 97859392 | rs234452 | 97890436 | rs2126439 | 98220423 | rs2370529 | 98263541 |
| 19 | 8 | 3 copies | 146 |  |  | pter |  | qter |  |  |  |
|  | 9 | 1 copy | 0.51 | kgp8717902 | 21846166 | rs7850937 | 21846285 | kgp18352303 | 22356957 | rs961831 | 22362104 |
|  | 9 | 0 copies | 0.22 | kgp22843691 | 21886934 | kgp22843691 | 21886934 | kgp22846305 | 22107121 | kgp18598392 | 22108328 |
| 20 | 4 | 3 copies | 0.32 | rs6836534 | 188230701 | rs1319774 | 188258978 | rs9992899 | 188583907 | rs12648438 | 188628654 |
|  | 6 | 3 copies | 171 |  |  | pter |  | qter |  |  |  |
|  | 6 | UPID | 21.7 | rs4706592 | 75876767 | rs4706592 | 75876767 | rs1206155 | 97549206 | rs1766531 | 97576033 |
|  | 7 | 1 copy | 0.15 | rs1012221 | 14364565 | rs17168073 | 14369455 | rs1431528 | 14524387 | rs12531250 | 14554546 |
|  | 8 | 1 copy | 0.77 | rs7825166 | 129215846 | rs2909246 | 129230194 | rs7829061 | 130000524 | rs1372454 | 130030130 |
|  | 9 | 3 copies | 141 |  |  | pter |  | qter |  |  |  |
|  | 9 | 1 copy | 2.29 | rs7860695 | 20021047 | rs7860695 | 20021047 | kgp18547864 | 22312174 | kgp4447392 | 22337197 |
|  | 9 | 0 copies | 0.43 | kgp18296111 | 21785142 | kgp18296111 | 21785142 | kgp18433344 | 22214069 | kgp18309277 | 22219049 |
|  | 19 | 3 copies | 59.1 |  |  | pter |  | qter |  |  |  |
|  | 21 | 3 copies | 48.1 |  |  | pter |  | qter |  |  |  |
|  | X | 0 copies | 0.89 | rs2071874 | 136842785 | rs5931209 | 136846042 | rs12006775 | 137738575 | rs657 | 137746181 |
| 21D | 9 | 1 copy | 1.35 | rs1869204 | 21690958 | rs6475552 | 21701674 | rs7019344 | 23051397 | rs12375567 | 23059458 |
|  | 9 | 0 copies | 0.18 | kgp18257202 | 21853264 | kgp18257202 | 21853264 | kgp8773938 | 22036112 | kgp2757377 | 22041155 |
|  | X | 0 copies | 1.09 | rs2073051 | 132473645 | rs10126402 | 132484049 | rs12687873 | 133572395 | rs12847109 | 133584837 |
| 21R | 8 | 1 copy | 66.1 |  |  | pter |  | cnvi0001815 | 66094238 | rs10094848 | 66139198 |
|  | 9 | 1 copy | 1.35 | rs1869204 | 21690958 | rs6475552 | 21701674 | rs7019344 | 23051397 | rs12375567 | 23059458 |
|  | 9 | 0 copies | 0.18 | kgp18257202 | 21853264 | kgp18257202 | 21853264 | kgp8773938 | 22036112 | kgp2757377 | 22041155 |
|  | 9 | 1 copy | 0.20 | cnvi0061697 | 137358557 | rs6537897 | 137394805 | rs4548258 | 137599444 | rs3109682 | 137628949 |
|  | 13 | 3 copies | 7.2 |  |  | cen |  | rs9634429 | 26676366 | rs1886455 | 26749216 |
|  | 13 | UPID | 19.2 | rs1886455 | 26749216 | rs1886455 | 26749216 | rs4941527 | 45985705 | rs11616734 | 45997346 |
|  | 13 | 1 copy | 69.1 | rs11616734 | 45997346 | rs11616734 | 45997346 | qter |  |  |  |
|  | 17 | 1 copy | 0.56 |  |  | pter |  | rs838371 | 556025 | rs2644714 | 579938 |
|  | 19 | 3 copies | 59.1 |  |  | pter |  | qter |  |  |  |
|  | 20 | 1 copy | 3.24 | rs1980576 | 57045667 | rs6070510 | 57134366 | rs6061832 | 60376611 | rs6089530 | 60439930 |
|  | X | 0 copies | 1.09 | rs2073051 | 132473645 | rs10126402 | 132484049 | rs12687873 | 133572395 | rs12847109 | 133584837 |
| 22 |  | NA |  |  |  |  |  |  |  |  |  |
| 23 | 9 | UPID | 32.9 |  |  | pter |  | rs12377462 | 32904589 | rs4879670 | 33195408 |
|  | 9 | 0 copies | 0.51 | SNP921907393 | 21907393 | SNP921907393 | 21907393 | SNP922419352 | 22419352 | SNP922421855 | 22421855 |
| 24 | 9 | 1 copy | 0.96 | rs4977686 | 21189680 | rs10125010 | 21206028 | rs2779747 | 22161484 | SNP922169676 | 22169676 |
|  | 9 | 0 copies | 0.16 | SNP921816187 | 21816187 | SNP921816187 | 21816187 | SNP921981106 | 21981106 | rs2811711 | 21983964 |
| 25 | 9 | UPID | 38.3 |  |  | pter |  | rs189315 | 38280691 | rs4878795 | 38376173 |
|  | 9 | 0 copies | 0.10 | kgp9393505 | 21895053 | kgp9393505 | 21895053 | kgp22848339 | 21993311 | rs2811711 | 21993964 |
| 26 | 3 | 3 copies | 1.17 | rs7628297 | 27127270 | rs1522166 | 27163310 | rs1599951 | 28334792 | rs4306817 | 28635630 |
|  | 5 | 1 copy | 74.6 | rs7706863 | 106284307 | rs4245987 | 106339147 | qter |  |  |  |
|  | 7 | 1 copy | 38.2 |  |  | pter |  | rs7806250 | 38198153 | rs2736948 | 38290010 |
|  | 7 | 3 copies | 16.5 | rs11761882 | 99419405 | rs6967487 | 99440127 | rs1049337 | 115987823 | rs1989795 | 116034060 |
|  | 7 | 1 copy | 16.9 | rs2855983 | 142178383 | rs11327 | 142210536 | qter |  |  |  |
|  | 9 | 1 copy | 2.50 | rs16936868 | 130446717 | rs12554024 | 130503867 | rs102993 | 133004893 | rs3739508 | 133129279 |
|  | 13 | 3 copies | 32.3 | rs2876794 | 82825903 | rs9546361 | 82849103 | qter |  |  |  |
|  | 19 | 1 copy | 1.58 | rs4802262 | 50841726 | rs7253390 | 50880466 | rs4804036 | 52459483 | rs4595896 | 52502740 |
|  | X | 3 copies | 0.35 | rs2738330 | 237940 | cnvi116483 | 320833 | rs6579607 | 667125 | rs4603065 | 701031 |
| 27 | 9 | 1 copy | 0.18 | kgp551113 | 21818674 | kgp18263827 | 21826187 | kgp4570145 | 22008026 | rs3217973 | 22009960 |
|  | 9 | 0 copies | 0.11 | kgp2975580 | 21900977 | kgp2975580 | 21900977 | kgp4570145 | 22008026 | rs3217973 | 22009960 |
|  | 10 | 1 copy | 0.42 | rs9418829 | 128865600 | rs7068057 | 128880582 | rs7069740 | 129300464 | rs12252756 | 129381096 |
|  | 13 | 1 copies | 0.08 | rs2804090 | 48982973 | rs17071686 | 48985639 | rs9332077 | 49064090 | rs9332054 | 49070345 |
|  | 21 | 1 copy | 0.06 | rs225327 | 43763206 | rs225334 | 43766560 | rs11909987 | 43822402 | rs11700858 | 43835855 |
| 28 | 4 | 1 copy | 0.33 | rs12650938 | 165683201 | rs4550872 | 165711918 | rs6847921 | 166039585 | rs17585396 | 166144008 |
|  | 5 | 1 copy | 0.25 | rs2964519 | 170714609 | rs2914331 | 170726247 | rs7729835 | 170971863 | rs1678787 | 171047194 |
|  | 9 | 1 copy | 0.79 | rs10116936 | 21277550 | rs4978097 | 21294921 | rs1547705 | 22082375 | rs1333040 | 22083404 |
|  | 9 | 0 copies | 0.45 | rs10811568 | 21543444 | rs10811568 | 21543444 | kgp22853822 | 21991106 | kgp22848339 | 21993311 |
|  | 14 | 1 copy | 0.27 | rs92233 | 98725340 | rs1892231 | 98734567 | rs1759072 | 99003134 | rs4900409 | 99056611 |
|  | 16 | 1 copy | 0.35 | rs4360931 | 61471926 | rs34730014 | 67516605 | rs11558534 | 67867739 | rs16957594 | 67935066 |
| 29 | 3 | 3 copies | 1.59 | rs7622634 | 175510715 | rs9884045 | 175658506 | rs1499880 | 177243737 | rs7630298 | 177448528 |
|  | 8 | 1 copy | 0.07 | rs16903937 | 129987132 | rs7008870 | 130002166 | rs4286912 | 130070676 | rs11784932 | 130095478 |
|  | 9 | 3 copies | 141 |  |  | pter |  | qter |  |  |  |
|  | 9 | 1 copy | 0.29 | rs3898946 | 20822078 | rs3898946 | 20822078 | rs1424859 | 21114982 | rs1016129 | 21122681 |
|  | 9 | 1 copy | 1.78 | rs7854222 | 21734348 | rs7854222 | 21734348 | rs274915 | 23513235 | rs655258 | 23542707 |
|  | 9 | 0 copies | 1.05 | kgp9789780 | 21782430 | kgp9789780 | 21782430 | rs6475687 | 22829231 | rs4369069 | 22834082 |
| 30 | 1 | 1 copy | 0.08 | rs11211481 | 47694167 | cnvi0054264 | 47698203 | cnvi0134340 | 47777455 | rs3925058 | 47797623 |
|  | 3 | 1 copy | 35.9 | rs2372689 | 44509710 | rs11714213 | 44524870 | rs6774035 | 80461975 | rs6791878 | 80493471 |
|  | 16 | 1 copy | 0.36 | rs9938207 | 6729357 | kgp22842416 | 6739989 | rs1382482 | 7103790 | rs7191854 | 7112003 |
| 31 | 3 | 3 copies | 104 |  |  | cen |  | qter |  |  |  |
|  | 5 | 3 copies | 39.3 |  |  | pter |  | rs7700754 | 39253022 | rs13179493 | 39426307 |
|  | 9 | 3 copies | 37.9 |  |  | pter |  | rs2025440 | 37889241 | rs2165776 | 38165646 |
| 32 | 9 | UPID | 47.3 |  |  | pter |  | cen |  |  |  |
|  | 9 | 0 copies | 0.15 | kgp18644213 | 21941196 | kgp18644213 | 21941196 | kgp18393910 | 22086767 | kgp364475 | 22087473 |
|  | 18 | 3 copies | 15.4 |  |  | pter |  | cen |  |  |  |
|  | X | 0 copies | 0.09 | rs4239965 | 47244180 | rs7055658 | 47252027 | rs2071780 | 47341821 | rs2071778 | 47343254 |
| 33 | 9 | UPID | 33.1 |  |  | pter |  | rs17326539 | 33111764 | rs10813958 | 33165985 |
|  | 9 | 0 copies | 0.17 | kgp6460326 | 21804693 | kgp6460326 | 21804693 | rs3731239 | 21974218 | kgp8063458 | 21979242 |
|  | 16 | 3 copies | 34.6 |  |  | pter |  | cen |  |  |  |
| 34 | 6 | 1 copy | 0.93 | rs17090905 | 156580748 | rs9384443 | 156560746 | cnvi0159118 | 157492559 | rs9347262 | 157868141 |
|  | 11 | 1 copy | 1.85 | rs11229895 | 59026901 | rs12796847 | 59118499 | rs582338 | 60972954 | rs3741265 | 61165280 |
| 35D | 9 | 1 copy | 1.26 | rs10811568 | 21533444 | rs10965063 | 21539638 | rs10125975 | 22795644 | rs9298841 | 22801336 |
|  | 9 | 0 copies | 0.56 | rs13297146 | 21616953 | rs13297146 | 21616953 | rs1095898 | 22179716 | SNP922180001 | 22180001 |
| 35R | 9 | 1 copy | 1.26 | rs10811568 | 21533444 | rs10965063 | 21539638 | rs10125975 | 22795644 | rs9298841 | 22801336 |
|  | 9 | 0 copies | 0.56 | rs13297146 | 21616953 | rs13297146 | 21616953 | rs1095898 | 22179716 | SNP922180001 | 22180001 |
| 36 |  | NC |  |  |  |  |  |  |  |  |  |
| 37 | 13 | UPID | 73.6 | rs11840294 | 40731921 | rs9532988 | 41547706 | qter |  |  |  |
| 38 | 4 | 1 copy | 0.11 | rs2107028 | 108968221 | rs4245928 | 108977666 | rs22851312 | 109086280 | rs2343113 | 109104682 |
|  | 7 | 1 copy | 17.1 | rs980385 | 141972370 | rs361429 | 142028118 | qter |  |  |  |
|  | 9 | 1 copy | 26.6 | rs6475464 | 20597576 | rs6475467 | 20638248 | cen |  |  |  |
|  | 9 | 0 copies | 0.55 | kgp18588676 | 21827192 | kgp18588676 | 21827192 | kgp18304005 | 22379145 | kgp9668928 | 22382738 |
|  | 10 | 1 copy | 1.37 | rs6586103 | 89584002 | rs12571737 | 89618914 | rs11203042 | 90989109 | rs7094601 | 91018520 |
|  | 11 | 3 copies | 33.9 |  |  | pter |  | rs3781578 | 33906826 | rs11032471 | 34006522 |
| 39 |  | NC |  |  |  |  |  |  |  |  |  |
| 40D | 1 | 1 copy | 0.07 | rs10890472 | 47708112 | rs10890473 | 47711456 | rs11211512 | 47777884 | rs3925058 | 47797623 |
|  | 6 | 3 copies | 15.6 | rs9345265 | 93378241 | rs6454911 | 93473885 | rs528400 | 109079791 | rs11153183 | 109817276 |
|  | 9 | UPID | 35.4 |  |  | pter |  | rs10972487 | 35482149 | rs2295842 | 35559213 |
|  | 9 | 0 copies | 0.26 | rs7037577 | 21772036 | rs7037577 | 21772036 | kgp18472633 | 22031052 | kgp5489791 | 22037071 |
|  | 13 | 1 copy | 0.09 | rs198604 | 48984063 | rs4151550 | 48985305 | rs9332054 | 49070345 | rs9332033 | 49078992 |
|  | 20 | 3 copies | 1.24 | rs96074300 | 11415133 | rs761682 | 11464227 | rs6134650 | 12708516 | rs6041604 | 12742546 |
| 41 | 1 | 1 copy | 0.07 | rs2245122 | 47645995 | rs11211484 | 47708369 | rs11211512 | 47777884 | rs11211516 | 47796110 |
|  | 9 | UPID | 23.9 |  |  | pter |  | rs2383296 | 23901927 | rs2218805 | 23938481 |
|  | 9 | 0 copies | 0.20 | kgp1815726 | 21793673 | kgp1815726 | 21793673 | kgp22848339 | 21993311 | kgp18684821 | 21988670 |
|  | 10 | 0 copies | 0.06 | rs1234221 | 89616479 | rs35161364 | 89630424 | rs17107001 | 89686509 | rs35917308 | 89690827 |
|  | 10 | 1 copy | 0.28 | rs1234221 | 89616479 | rs35161364 | 89630424 | rs10887791 | 89909218 | rs868872 | 89950581 |
|  | 11 | 1 copy | 0.19 | rs7933499 | 33903328 | rs911817 | 33908547 | rs1004787 | 34097254 | rs17699517 | 34100008 |
| 42 | 1 | 1 copy | 0.07 | rs2249636 | 47686533 | cnvi0054264 | 47698203 | rs6697298 | 47766943 | rs3925058 | 47797623 |
|  | 9 | UPID | 36.5 |  |  | pter |  | rs1962524 | 36485202 | rs7873864 | 36740957 |
|  | 9 | 0 copies | 3.00 | rs7025014 | 19562817 | rs7025014 | 19562817 | kgp5070770 | 22562121 | kgp22852883 | 22579889 |
|  | 19 | UPID | 14.1 |  |  | pter |  | rs12019 | 14072734 | rs1982632 | 14139004 |
| 43 | 6 | 1 copy | 30.5 | rs2145546 | 72415931 | rs829473 | 72440951 | rs601907 | 102922642 | rs9322668 | 103071742 |
|  | 9 | UPID | 33.6 |  |  | pter |  | rs7033200 | 33565358 | rs7036030 | 34085859 |
|  | 9 | 0 copies | 0.19 | kgp18391916 | 21812552 | kgp18391916 | 21812552 | rs2811712 | 21998035 | rs3218005 | 22000247 |
| 44 | 3 | 1 copy | 3.53 | rs6809907 | 137676846 | rs13099679 | 137878882 | rs17195428 | 141409701 | rs16851740 | 141469631 |
|  | 9 | 1 copy | 26.4 |  |  | pter |  | rs16910426 | 26403617 | rs4576502 | 26463375 |
|  | 9 | 0 copies | 2.31 | rs7033704 | 21051966 | rs7033704 | 21051966 | rs2210292 | 23361805 | rs4336697 | 23373293 |
|  | 9 | 1 copy | 0.47 | rs1342018 | 75782131 | rs17058735 | 75922205 | rs2125391 | 76389514 | rs1458490 | 76412152 |
|  | 9 | 1 copy | 28.8 | rs12555718 | 80874338 | rs3758200 | 80918333 | rs17785570 | 109691521 | rs11532981 | 109752970 |
| 45 | 11 | 1 copy | 15.1 | rs1943699 | 84536635 | rs12274130 | 84561299 | rs947979 | 99634866 | rs1301846 | 99666292 |
| 46 | 1 | 1 copy | 0.06 | cnvi0054261 | 47697907 | rs10890472 | 47708112 | rs3125630 | 47767914 | rs11211516 | 47796110 |
|  | 5 | 3 copies | 46.1 |  |  | pter |  | cen |  |  |  |
|  | 7 | 3 copies | 75.4 | rs2527040 | 8368148 | rs10488266 | 83701677 | qter |  |  |  |
|  | 9 | UPID | 33.0 |  |  | pter |  | rs3818644 | 33025113 | rs544723 | 33103173 |
|  | 9 | 0 copies | 2.77 | rs9792640 | 20035279 | rs9792640 | 20035279 | rs1448779 | 22806356 | rs9298841 | 22811363 |
| 47D | 4 | 1 copy | 0.30 | rs4245926 | 108967846 | rs4245927 | 108969590 | rs9307319 | 109274125 | rs12374269 | 109288854 |
|  | 9 | 1 copy | 0.19 | kgp10384822 | 21854159 | kgp22847279 | 21856524 | kgp3764974 | 22043612 | kgp9423146 | 22048683 |
|  | 17 | UPID | 52.7 | rs614334 | 27904985 | rs4494608 | 28488791 | qter |  |  |  |
| 47R | 17 | UPID | 49.9 | rs 2640837 | 30990750 | rs2188953 | 31319024 | qter |  |  |  |
| U1D | 6 | 3 copies | 0.31 | rs3799070 | 70020740 | rs12211885 | 70201287 | rs3778241 | 70512716 | rs4304126 | 70569488 |
|  |  | 1 copy | 34.4 | rs7739096 | 79215666 | rs1180829 | 79477495 | rs2049923 | 113895049 | rs9374411 | 114079343 |
|  |  | 1 copy | 0.49 | rs4946053 | 115420216 | rs238589 | 115470639 | rs 12660488 | 115965140 | rs763229 | 115971864 |
|  |  | 1 copy | 2.62 | rs 6568951 | 117038717 | rs4946188 | 117079875 | rs195077 | 119696844 | rs2357524 | 119928027 |
|  |  | 1 copy | 1.30 | rs1267992 | 124328325 | rs1510298 | 124344877 | rs1260731 | 125649002 | rs1353197 | 125818032 |
|  |  | 1 copy | 0.47 | rs17055628 | 128677748 | rs9402035 | 128702994 | rs1508453 | 129171964 | rs 6569559 | 129183019 |
|  |  | 1 copy | 3.10 | rs6937012 | 130988748 | rs9388825 | 131033848 | rs1208228 | 134133659 | rs12203224 | 134143187 |
|  |  | 1 copy | 3.12 | rs6904753 | 137015976 | rs1009709 | 137059442 | rs12664111 | 140181508 | rs6903961 | 140195104 |
|  |  | 1 copy | 1.37 | rs7773151 | 142385102 | rs4896574 | 142439353 | rs6929191 | 143807471 | rs161064 | 143838026 |
|  |  | 1 copy | 7.26 | rs2275345 | 143914653 | rs954349 | 143977500 | rs2073069 | 151238363 | rs4869953 | 151251690 |
|  |  | 1 copy | 0.47 | rs4869993 | 151515023 | rs752104 | 151523057 | rs7751941 | 151988351 | rs6900157 | 151995820 |
|  |  | 1 copy | 3.67 | rs214955 | 152739399 | rs214976 | 152813957 | rs11156080 | 156481213 | rs2354393 | 156497361 |
|  |  | 1 copy | 3.68 | rs10485205 | 157238666 | rs176390 | 157274781 | rs7761377 | 160952471 | rs7759633 | 160990980 |
|  |  | 1 copy | 6.24 | rs910727 | 161477997 | rs13197494 | 161512964 | rs9457335 | 167752761 | rs4708591 | 167805247 |
|  |  | 1 copy | 0.78 | rs9477987 | 169656143 | rs7752938 | 169723045 | rs9348316 | 170507358 | rs9460003 | 170549797 |
|  | 9 | 1 copy | 0.07 | rs4478653 | 21843221 | rs4977569 | 21884495 | rs7041637 | 21951866 | rs3731239 | 21964218 |
|  |  | 0 copies | 0.01 | rs3731239 | 21964 218 | rs3731239 | 21964218 | rs373217 | 21974661 | rs2811712 | 21988 035 |
| U1R | 4 | 1 copy | 19.2 | rs870569 | 157493751 | rs13123652 | 157592697 | rs11133106 | 176744502 | rs6553892 | 176826574 |
|  | 6 | 3 copies | 0.31 | rs3799070 | 70020740 | rs12211885 | 70201287 | rs3778241 | 70512716 | rs4304126 | 70569488 |
|  |  | 1 copy | 34.4 | rs7739096 | 79215666 | rs1180829 | 79477495 | rs2049923 | 113895049 | rs9374411 | 114079343 |
|  |  | 1 copy | 0.49 | rs4946053 | 115420216 | rs238589 | 115470639 | rs 12660488 | 115965140 | rs763229 | 115971864 |
|  |  | 1 copy | 2.62 | rs 6568951 | 117038717 | rs4946188 | 117079875 | rs195077 | 119696844 | rs2357524 | 119928027 |
|  |  | 1 copy | 1.30 | rs1267992 | 124328325 | rs1510298 | 124344877 | rs1260731 | 125649002 | rs1353197 | 125818032 |
|  |  | 1 copy | 0.47 | rs17055628 | 128677748 | rs9402035 | 128702994 | rs1508453 | 129171964 | rs 6569559 | 129183019 |
|  |  | 1 copy | 3.10 | rs6937012 | 130988748 | rs9388825 | 131033848 | rs1208228 | 134133659 | rs12203224 | 134143187 |
|  |  | 1 copy | 3.12 | rs6904753 | 137015976 | rs1009709 | 137059442 | rs12664111 | 140181508 | rs6903961 | 140195104 |
|  |  | 1 copy | 1.37 | rs7773151 | 142385102 | rs4896574 | 142439353 | rs6929191 | 143807471 | rs161064 | 143838026 |
|  |  | 1 copy | 7.26 | rs2275345 | 143914653 | rs954349 | 143977500 | rs2073069 | 151238363 | rs4869953 | 151251690 |
|  |  | 1 copy | 0.47 | rs4869993 | 151515023 | rs752104 | 151523057 | rs7751941 | 151988351 | rs6900157 | 151995820 |
|  |  | 1 copy | 3.67 | rs214955 | 152739399 | rs214976 | 152813957 | rs11156080 | 156481213 | rs2354393 | 156497361 |
|  |  | 1 copy | 3.68 | rs10485205 | 157238666 | rs176390 | 157274781 | rs7761377 | 160952471 | rs7759633 | 160990980 |
|  |  | 1 copy | 6.24 | rs910727 | 161477997 | rs13197494 | 161512964 | rs9457335 | 167752761 | rs4708591 | 167805247 |
|  |  | 1 copy | 0.78 | rs9477987 | 169656143 | rs7752938 | 169723045 | rs9348316 | 170507358 | rs9460003 | 170549797 |
|  | 9 | 1 copy | 0.07 | rs4478653 | 21843221 | rs4977569 | 21884495 | rs7041637 | 21951866 | rs3731239 | 21964218 |
|  |  | 0 copies | 0.01 | rs3731239 | 21964 218 | rs3731239 | 21964218 | rs373217 | 21974661 | rs2811712 | 21988 035 |
| U2 | 9 | UPID | 34.2 | rs2260055 | 74832 | rs552848 | 228389 | rs10119711 | 34397204 | rs11547035 | 34449025 |
|  |  | 1 copy | 0.16 | rs4345650 | 21807777 | rs7867176 | 21815996 | rs3731239 | 21964218 | rs2811709 | 21970151 |
|  |  | 0 copies | 0.005 | rs2811709 | 21970151 | rs2811709 | 21970151 | rs3731217 | 21974661 | rs3218020 | 21987872 |
|  |  | 3 copies | 0.49 | rs2900142 | 119871703 | rs1999009 | 119920460 | rs1335247 | 120414392 | rs10818205 | 120437033 |
|  | 15 | 1 copy | 0.29 | rs10467975 | 40884944 | rs6493067 | 40931290 | rs2176870 | 41223702 | rs11070396 | 41310214 |
| U2R | 6 | 3 copies | 45.7 | rs13196989 | 184373 | rs13196989 | 184373 | pter |  |  |  |
|  | 9 | UPID | 34.2 | rs2260055 | 74832 | rs552848 | 228389 | rs10119711 | 34397204 | rs11547035 | 34449025 |
|  |  | 3 copies | 0.49 | rs2900142 | 119871703 | rs1999009 | 119920460 | rs1335247 | 120414392 | rs10818205 | 120437033 |
|  | 15 | 1 copy | 0.29 | rs10467975 | 40884944 | rs6493067 | 40931290 | rs2176870 | 41223702 | rs11070396 | 41310214 |
| U4 | 4 | 3 copies | 0.08 | 61955 | rs2131464 | 28404856 | rs10805279 | 28410302 | rs7698028 | 28472257 | rs10049756 |
|  | 9 | UPID | 21.6 | rs3008170 | 192118 | rs636922 | 205269 | rs7023329 | 21806528 | rs1544195 | 21820479 |
|  |  | 0 copies | 0.15 | rs1544195 | 21820479 | rs1544195 | 21820479 | rs3731217 | 21974661 | rs3218020 | 21987872 |
|  | 10 | 1 copy | 1.02 | rs1022427 | 89596169 | rs1234220 | 89635453 | rs717576 | 90657573 | rs1372325 | 90683549 |
|  | 13 | 1 copy | 0.05 | rs9593799 | 82996585 | rs92531462 | 83001708 | rs9805405 | 83055928 | rs1333072 | 83063672 |

cen, centromere; Chr, chromosome; D, diagnosis; FAS, first abnormal SNP; FNS, first normal SNP; LAS, last abnormal SNP; LNS, last normal SNP; NA, not analyzed; NC, no changes; Pos, position based on *homo sapiens* high coverage assembly GRCh37; pter, terminal of p-arm; qter, terminal of q-arm; R, relapse; SNP, single nucleotide polymorphism; UPID, uniparental isodisomy.

^a^Distance between the FAS and the LAS.
