# Supplementary material for: Deep sequencing and SNP array analyses of pediatric T-cell acute lymphoblastic leukemia reveal NOTCH1 mutations in minor subclones and a high incidence of uniparental isodisomies affecting CDKN2A
Source: J Hematol Oncol. 2015 Apr 24;8:42. doi: 10.1186/s13045-015-0138-0 (PMC4412034; doi:10.1186/s13045-015-0138-0)
Supplement: Additional file 3: Table S3. — Detailed information on the 46 identified mutations. [file 13045_2015_138_MOESM3_ESM.doc]

**Additional file 3: Table S3.** Detailed information on the 46 identified mutations.

| Gene | Case  No. | Position^a^ | Ref^b^ | Var^b^ | No. of  var/total^b^ | VAF | Score^b^ | Accession nr^c^ | Nucleotide  substitution | Amino acid  substitution | Previously  reported in  T-ALL^e^ |
| --- | --- | --- | --- | --- | --- | --- | --- | --- | --- | --- | --- |
| *BCL11B* | 23 | 14:99641825 | T | C | 21/60 | 0.35 | 483 | NP_612808 | c.1843T>C^d^ | p.T450A | No |
| *CREBBP* | 40 | 16:3778424 | T | G | 45/89 | 0.51 | 1000 | NP_004371.2 | c.6624A>C | p.Q2208H | Yes |
| *CREBBP* | 18 | 16:3827658 | T | C | 209/408 | 0.51 | 1000 | NP_004371.2 | c.2114A>G | p.N705S | No |
| *DNMT3A* | 8 | 2:25470516 | G | A | 206/441 | 0.47 | 1000 | NP_783328.1 | c.958C>T | p.R320X | No |
| *EZH2* | 9 | 7:148513776 | C | G | 84/188 | 0.45 | 1000 | NP_694543.1 | c.1505G>C^d^ | p.R502P | No |
| *FBXW7* | 8 | 4:153247168 | T | C | 123/279 | 0.44 | 1000 | NP_361014.1 | c.1634A>G | p.Y545C | No |
| *FBXW7* | 14 | 4:153247289 | G | A | 230/614 | 0.37 | 1000 | NP_361014.1 | c.1513C>T | p.R505C | Yes |
| *FBXW7* | 16 | 4:153247289 | G | A | 92/675 | 0.14^f^ | 1000 | NP_361014.1 | c.1513C>T | p.R505C | Yes |
| *FBXW7* | 32 | 4:153247289 | G | A | 236/519 | 0.45 | 1000 | NP_361014.1 | c.1513C>T | p.R505C | Yes |
| *FBXW7* | 9 | 4:153249384 | CG | AC | 165/310 | 0.53 | 1000 | NP_361014.1 | c.1393_1394CG>AC^d^ | p.R465V | No |
| *FBXW7* | 19 | 4:153249384 | C | T | 163/336 | 0.49 | 1000 | NP_361014.1 | c.1394G>A | p.R465H | Yes |
| *FBXW7* | 20 | 4:153249385 | G | A | 190/422 | 0.45 | 1000 | NP_361014.1 | c.1393C>T | p.R465C | Yes |
| *FBXW7* | 28 | 4:153249385 | G | A | 253/530 | 0.48 | 1000 | NP_361014.1 | c.1393C>T | p.R465C | Yes |
| *FBXW7* | 35 | 4:153249385 | G | A | 236/562 | 0.42 | 1000 | NP_361014.1 | c.1393C>T | p.R465C | Yes |
| *FBXW7* | 36 | 4:153249385 | G | A | 184/487 | 0.38 | 1000 | NP_361014.1 | c.1393C>T | p.R465C | Yes |
| *FBXW7* | 9 | 4:153249457 | G | A | 155/346 | 0.45 | 1000 | NP_361014.1 | c.1312C>T | p.R441W | No |
| *FBXW7* | 23 | 4:153250856 | TGT | --- | 39/339 | 0.12^f^ | 356 | NP_361014.1 | c.1202_1204delACA | p.N401delN | No |
| *JAK1* | 32 | 1:65310517 | C | T | 185/431 | 0.43 | 1000 | NP_002218.2 | c.2171G>A^d^ | p.R724H | Yes |
| *JAK3* | 32 | 19:17949108 | C | T | 90/189 | 0.48 | 1000 | NP_000206.2 | c.1533G>A | p.M511I | Yes |
| *NOTCH1* | 41 | 9:139390716 | G | T | 34/262 | 0.13^f^ | 270 | NP_060087.3 | c.7475C>A | p.S2492X | Yes |
| *NOTCH1* | 25 | 9:139390813 | C | A | 65/123 | 0.53 | 1000 | NP_060087.3 | c.7378G>T | p.E2460X | No |
| *NOTCH1* | 26 | 9:139397639 | A | T | 105/181 | 0.58 | 1000 | NP_060087.3 | c.5162T>A | p.V1721E | Yes |
| *NOTCH1* | 8 | 9:139397675 | A | G | 22/216 | 0.10^f^ | 83 | NP_060087.3 | c.5126T>C | p.L1709P | Yes |
| *NOTCH1* | 15 | 9:139397762 | A | T | 72/147 | 0.49 | 1000 | NP_060087.3 | c.5039T>A | p.I1680N | Yes |
| *NOTCH1* | 25 | 9:139397768 | A | G | 13/105 | 0.12^f^ | 131 | NP_060087.3 | c.5033T>C | p.L1678P | Yes |
| *NOTCH1* | 35 | 9:139397768 | A | G | 54/142 | 0.38 | 1000 | NP_060087.3 | c.5033T>C | p.L1678P | Yes |
| *NOTCH1* | 44 | 9:139397768 | A | G | 47/139 | 0.34 | 1000 | NP_060087.3 | c.5033T>C | p.L1678P | Yes |
| *NOTCH1* | 28 | 9:139399324 | --- | TTC | 15/59 | 0.25 | 324 | NP_060087.3 | c.4817_4818insGGA | p.F1606>LD | Yes |
| *NOTCH1* | 45 | 9:139399332 | AC | TG | 44/99 | 0.44 | 1000 | NP_060087.3 | c.4810_4811AC>TG^d^ | p.V1604Q | No |
| *NOTCH1* | 42 | 9:139399350 | C | G | 6/60 | 0.10^f^ | 22 | NP_060087.3 | c.4793G>C | p.R1598P | Yes |
| *NOTCH1* | 47 | 9:139399350 | C | G | 10/73 | 0.14 | 141 | NP_060087.3 | c.4793G>C | p.R1598P | Yes |
| *NOTCH1* | 38 | 9:139399356 | A | T | 19/77 | 0.25 | 408 | NP_060087.3 | c.4787T>A | p.L1596H | Yes |
| *NOTCH1* | 23 | 9:139399365 | A | G | 6/52 | 0.12^f^ | 13 | NP_060087.3 | c.4778T>C | p.L1593P | Yes |
| *NOTCH1* | 42 | 9:139399389 | A | G | 7/53 | 0.13^f^ | 29 | NP_060087.3 | c.4754T>C | p.L1585P | Yes |
| *NOTCH1* | 33 | 9:139399397 | CGG | --- | 14/46 | 0.30 | 348 | NP_060087.3 | c.4744_4746delCCG | p.P1582delP | Yes |
| *NOTCH1* | 25 | 9:139399409 | CAC | --- | 4/32 | 0.13^f^ | 18 | NP_060087.3 | c.4732_4734delGTG | p.V1578delV | Yes |
| *NOTCH1* | 24 | 9:139399410 | A | T | 13/38 | 0.34 | 282 | NP_060087.3 | c.4733T>A | pV1578G | Yes |
| *NOTCH1* | 32 | 9:139399422 | A | G | 27/56 | 0.48 | 661 | NP_060087.3 | c.4721T>C | p.L1547P | Yes |
| *NRAS* | 26 | 1:115258748 | C | T | 204/442 | 0.46 | 1000 | NP_002515.1 | c.34G>A | p.G12S | Yes |
| *NRAS* | 41 | 1:115258748 | C | T | 248/649 | 0.38 | 1000 | NP_002515.1 | c.34G>A | p.G12S | Yes |
| *PHF6* | 34 | X:133551319 | C | T | 222/227 | 0.98 | 529 | NP_115834.1 | c.955C>T | p.R319X | Yes |
| *PIK3CA* | 19 | 3:178952085 | A | G | 131/390 | 0.34 | 1000 | NP_006209.2 | c.3140A>G | p.H1047R | No |
| *PTEN* | 43 | 10:89717683 | C | G | 82/245 | 0.33 | 1000 | NP_000305.3 | c.708C>G^d^ | p.D236E | No |
| *PTEN* | 23 | 10:89720726 | G | T | 59/339 | 0.17^f^ | 1000 | NP_000305.3 | c.877G>T^d^ | p.G293X | No |
| *SETD2* | 7 | 3:47144879 | C | T | 134/426 | 0.31 | 1000 | NP_054878.5 | c.4874G>A^d^ | p.R1625H | No |
| *TCF3* | 37 | 19:1650225 | G | A | 33/83 | 0.40 | 903 | NP_003191.1 | c.23C>T | p.A8V | No |

Ref; reference; VAF, variant allele frequency; Var, variant.

^a^Based on positions from the GRCh37 genome build (http://www.ensembl.org/Homo_sapiens /Info/Index).

^b^According to the GeneSpring GX software (Agilent).

^c^Reference sequence available at http://www.ncbi.nlm.nih.gov/refseq/.

^d^Verified by Sanger sequencing and not present in remission samples.

^e^According to COSMIC database (cancer.sanger.ac.uk/).

^f^Considered as minor subclones based on the presence of other mutations or copy number abnormalities/segmental uniparental isodisomies at higher frequencies in the sample.
