# Supplementary material for: Deep sequencing and SNP array analyses of pediatric T-cell acute lymphoblastic leukemia reveal NOTCH1 mutations in minor subclones and a high incidence of uniparental isodisomies affecting CDKN2A
Source: J Hematol Oncol. 2015 Apr 24;8:42. doi: 10.1186/s13045-015-0138-0 (PMC4412034; doi:10.1186/s13045-015-0138-0)
Supplement: Additional file 4: Table S4. — Genetic features of diagnostic and relapse samples. [file 13045_2015_138_MOESM4_ESM.docx]

**Additional file 4: Table S4.** Genetic features of diagnostic and relapse samples

| Case  No. | Time to relapse (months) | Karyotype/  Imbalances/UPIDs identified by SNP array analysis | TCR  (FISH) | Sequencing | Genomic relationship between D and R |
| --- | --- | --- | --- | --- | --- |
| 4D |  | 46,XY^a^  del(9)(p21.3p21.3)x2,UPID(9)(p13.3pter) | *TRB* (62%) | NA |  |
| 4R1 | 4 | 46,XY  No changes identified by SNP array^b^ | *TRB* (11%) | NA | Not informative |
| 4R2 | 5 | 46,XY,t(7;11)(q35;p13)/46,idem,del(6)(q21q25),add(17)(p13)^a^  SNP array analysis not performed | *TRB* (58%) | NA | Clonal evolution |
| 6D |  | 46,XY,del(6)(q13q21)^a^  del(6)(q13q16.3),del(9)(p21.3p21.3)x2,UPID(9)(p21.1pter) | No | No mutations |  |
| 6R | 16 | 46,XY,del(6)(q13q21)  del(2)(p16.3p16.3),del(6)(q14.1q22.31),del(9)(p21.3p21.3)x2,  UPID(9)(p21.1pter) | No | NA | Ancestral clone |
| 10D |  | 46,XY  del(9)(p21.3p21.3)x2,UPID(10)(q21.3qter) | No | No mutations |  |
| 10R | 5 | 46,XY  No changes identified by SNP array^b^ | No | No mutations | Not informative |
| 15D |  | 46,XY^a^  dup(8)(q24.21q24.21),del(9)(p21.3p21.3)x2 | *TRA/D* (17%) | *NOTCH1* (p.I1680N) |  |
| 15R1 | 27 | 46,XY  del(8)(q21.3q21.3),dup(8)(q24.21q24.21),del(9)(p21.3p21.3)x2 | No | *NOTCH1* (p.I1680N) | Ancestral clone |
| 15R2 | 52 | 46,XY,del(6)(p12),der(17)t(6;17)(p12;p11),del(20)(q11)^a^  SNP array analysis not performed | No | NA | Ancestral clone |
| 21D |  | 46,XY^a^  del(X)(q26.2q26.2),del(9)(p21.3p21.3)x2 | No | No mutations |  |
| 21R | 29 | 47,XY,−8,−13,?der(17)t(13;17)(q1?;p1?),+19,+2mar^a^  del(X)(q26.2q26.2),del(8)(pterq13.1),del(9)(p21.3p21.3)x2,del(9)  (q34.2q34.3),dup(13)(q10q14.13),UPID(13)(q12.13q14.13),del(13)  (q14.13qter),del(17)(p13.3pter),+19,del(20)(q13.32q13.33) | No | NA | Clonal evolution |
| 29D |  | 47,XY,+9^a^  dup(3)(q26.32q26.32),del(8)(q24.1q24.1),+9,del(9)(p21.3p21.3)x3 | No | NA |  |
| 29R | 2 | 46,XY  SNP array analysis not performed | NA | NA | Not informative |
| 35D |  | ??,X?,del(9)(p21p21)x2^a^  del(9)(p21.3p21.3)x2 | NA | *FBXW7* (p.R465C), *NOTCH1* (p.L1678P) |  |
| 35R | 5 | 46,XX,del(9)(p21p21)x2  del(9)(p21.3p21.3)x2 | NA | NA | Identical clones |
| 40D |  | 46,XX  del(1)(p33p33),dup(6)(q16.1q21),del(9)(p21.3p21.3)x2,UPID(9)  (p13.3pter),del(13)(q14.2q14.2),dup(20)(p12.1p12.2) | No | *CREBBP* (p.Q2208H) |  |
| 40R | 7 | NA  SNP array analysis not performed | NA | NA | Not informative |
| 47D |  | 46,XX^a^  del(4)(q25q25),del(9)(p21.3p21.3),UPID(17)(q11.2qter) | No | *NOTCH1* (p.R1598P) |  |
| 47R | 13 | NA  UPID(17)(q11.2qter) | NA | No mutations | Ancestral clone |
| U1D |  | 46,XY  dup(6)(q13q13),del(6)(q14.1qter)^c^,del(9)(p21.3p21.3)x2 | NA | NA |  |
| U1R | 12 | NA  del(4)(q32.1q34.2),dup(6)(q13q13),del(6)(q14.1qter)^c^,del(9)(p21.3p21.3)x2 | NA | NA | Clonal evolution |
| U2D |  | 46,XX  del(9)(p21.3p21.3)x2,UPID(9)(p13.1pter),dup(9)(q33.1q33.1),  del(15)(q15.1q15.2) | NA | NA |  |
| U2R | 7 | NA  dup(6)(p21.1pter),UPID(9)(p13.3pter),dup(9)(q33.1q33.1),  del(15)(q15.1q15.2) | NA | NA | Ancestral clone |
| U3D |  | NA  UPID(9)(p13.1pter),del(9)(p21.3p21.3)x2,dup(17)(q12qter) | NA | NA |  |
| U3R | NK | NA  No changes identified by SNP array^b^ | NA | NA | Not informative |
| U4D |  | 46,XY  dup(4)(p15.1p15.1),del(9)(p21.3p21.3)x2,UPID(9)(p21.3pter),  del(10)(q23.31q23.31),del(13)(q31.1q31.1) | NA | NA |  |
| U4R | 11 | NA  dup(4)(p15.1p15.1),del(9)(p21.3p21.3)x2,UPID(9)(p21.3pter),  del(10)(q23.31q23.31),del(13)(q31.1q31.1) | NA | NA | Identical clones |

D, diagnosis; FISH, fluorescence *in situ* hybridization; NA, not analyzed; NK, not known; R, relapse; SNP, single nucleotide polymorphism; TCR, T-cell receptor rearrangement; UPID, uniparental isodisomy.

^a^These karyotypes have previously been reported [[1-4](#_ENREF_1)].

^b^Most likely low tumor cell count because there were no signs of TCR rearrangements or other clonal changes in the SNP array analysis.

^c^This deletion was quite complex with 28 breakpoints between 6q14 and 6qter and with copy number changes oscillating between one and two copies, thus representing chromothripsis.

**References**

1. Heim S, Békassy AN, Garwicz S, Heldrup J, Kristoffersson U, Mandahl N et al. Bone marrow karyotypes in 94 children with acute leukemia. Eur J Haematol. 1990;44:227-33.

2. Andreasson P, Höglund M, Békassy AN, Garwicz S, Heldrup J, Mitelman F et al. Cytogenetic and FISH studies of a single center consecutive series of 152 childhood acute lymphoblastic leukemias. Eur J Haematol. 2000;65:40-51.

3. Karrman K, Isaksson M, Paulsson K, Johansson B. The insulin receptor substrate 4 gene (*IRS4*) is mutated in paediatric T-cell acute lymphoblastic leukaemia. Br J Haematol. 2011;155:516-9.

4. Olsson L, Castor A, Behrendtz M, Biloglav A, Forestier E, Paulsson K et al. Deletions of *IKZF1* and *SPRED1* are associated with poor prognosis in a population-based series of pediatric B-cell precursor acute lymphoblastic leukemia diagnosed between 1992 and 2011. Leukemia. 2014;28:302-10.
