# Supplementary material for: Deep sequencing and SNP array analyses of pediatric T-cell acute lymphoblastic leukemia reveal NOTCH1 mutations in minor subclones and a high incidence of uniparental isodisomies affecting CDKN2A
Source: J Hematol Oncol. 2015 Apr 24;8:42. doi: 10.1186/s13045-015-0138-0 (PMC4412034; doi:10.1186/s13045-015-0138-0)
Supplement: Additional file 5: Table S5. — Survival in relation to clinical and genetic features. [file 13045_2015_138_MOESM5_ESM.docx]

**Additional file 5: Table S5.** Survival in relation to clinical and genetic features

| Parameters | No. of  cases | pEFS (SE) | *P* value^a^ | pOS (SE) | *P* value^a^ | pRFS (SE) | *P* value^a^ |
| --- | --- | --- | --- | --- | --- | --- | --- |
| Total cohort | 47 | 0.604 (0.07) |  | 0.665 (0.07) |  | 0.815 (0.06) |  |
| Clinical features |  |  |  |  |  |  |  |
| Age (<10 years *vs*. ≥10 years) | 27/20 | 0.60 (0.10) *vs*. 0.60 (0.10) | 0.72 | 0.68 (0.10) *vs*. 0.65 (0.11) | 0.62 | 0.74 (0.09) *vs.* 0.94 (0.06) | 0.16 |
| Gender (male *vs*. female) | 38/9 | 0.67 (0.08) *vs*. 0.33 (0.16) | **0.02** | 0.75 (0.16) *vs*. 0.33 (0.16) | **0.003** | 0.82 (0.07) *vs.* 0.86 (0.13) | 0.94 |
| WBC count (<200 x 10^9^/l *vs*. ≥200 x 10^9^/l) | 30/17 | 0.67 (0.09) *vs*. 0.46 (0.13) | 0.47 | 0.77 (0.08) *vs*. 0.46 (0.13) | 0.13 | 0.85 (0.07) *vs.* 0.71 (0.15) | 0.67 |
| FISH results |  |  |  |  |  |  |  |
| TCR (wt *vs*. translocation) | 23/8 | 0.52 (0.11) *vs*. 0.42 (0.20) | 0.71 | 0.61 (0.11) *vs*. 0.42 (0.20) | 0.45 | 0.79 (0.10) *vs.* 0.57 (0.25) | 0.60 |
| Array findings |  |  |  |  |  |  |  |
| No. of aberrations^b^ (<3 *vs*. ≥3) | 25/15 | 0.54 (0.10) *vs*. 0.60 (0.13) | 0.92 | 0.66 (0.10) *vs*. 0.60 (0.13) | 0.33 | 0.74 (0.10) *vs.* 0.83 (0.11) | 0.75 |
| *CDKN2A* (wt *vs*. deletion) | 11/29 | 0.73 (0.14) *vs*. 0.48 (0.10) | 0.29 | 0.91 (0.09) *vs*. 0.52 (0.10) | **0.04** | 1.0 *vs.* 0.69 (0.10) | 0.08 |
| *CDKN2A* deletion (with *vs.*  without sUPID9p) | 12/17 | 0.40 (0.17) *vs.* 0.52 (0.12) | 0.68 | 0.40 (0.17) vs. 0.58 (0.12) | 0.48 | 0.60 (0.20) vs. 0.71 (0.12) | 0.74 |
| *STIL* (wt *vs*. deletion) | 35/5 | 0.56 (0.09) *vs*. 0.60 (0.22) | 0.85 | 0.64 (0.08) *vs*. 0.60 (0.22) | 0.49 | 0.78 (0.08) *vs.* 0.75 (0.22) | 0.66 |
| Gene involvement |  |  |  |  |  |  |  |
| *NOTCH1* (wt *vs*. mutation) | 23/16 | 0.69 (0.10) *vs*. 0.45 (0.14) | 0.19 | 0.77 (0.09) *vs*. 0.52 (0.14) | 0.12 | 0.86 (0.08) *vs.* 0.77 (0.15) | 0.76 |
| *FBXW7* (wt *vs*. mutation) | 28/11 | 0.49 (0.10) *vs*. 0.82 (0.12) | 0.11 | 0.55 (0.10) *vs*. 0.91 (0.09) | 0.06 | 0.79 (0.10) *vs.* 0.90 (0.10) | 0.56 |
| *NOTCH1* and/or *FBXW7*  (wt *vs*. mutation) | 17/22 | 0.56 (0.13) *vs*. 0.61 (0.11) | 0.73 | 0.68 (0.12) *vs*. 0.66 (0.10) | 0.90 | 0.80 (0.10) *vs.* 0.87 (0.09) | 0.51 |
| Epigenetic regulation^c^  (wt *vs*. mutation/deletion) | 32/7 | 0.60 (0.10) *vs*. 0.43 (0.19) | 0.28 | 0.70 (0.09) *vs*. 0.54 (0.20) | 0.43 | 0.84 (0.08) *vs.* 0.83 (0.15) | 0.87 |
| Signaling transduction^d^ (wt *vs*. mutation/deletion) | 31/8 | 0.59 (0.09) *vs*. 0.63 (0.17) | 0.92 | 0.68 (0.09) *vs*. 0.64 (0.17) | 0.47 | 0.81 (0.08) *vs.* 1.0 | 0.30 |

pEFS, probability of event-free survival (5 years); pOS, probability of overall survival (5 years); pRFS, probability of relapse-free survival (5 years); SE, standard error; TCR, T-cell receptor; WBC, white blood cell; wt, wild type.

^a^Significant *P* values are denoted in bold type.

^b^Copy number abnormalities and segmental uniparental isodisomies combined.

^c^*CREBBP*, *DNMT3A*, *EZH2, PHF6*, or *SETD2* mutations or deletions.

^d^*JAK1*, *JAK3*, *NRAS*, *PI3KCA*, or *PTEN* mutations or deletions.
