# Supplementary figures and images for: Deep sequencing and SNP array analyses of pediatric T-cell acute lymphoblastic leukemia reveal NOTCH1 mutations in minor subclones and a high incidence of uniparental isodisomies affecting CDKN2A
Source: J Hematol Oncol. 2015 Apr 24;8:42. doi: 10.1186/s13045-015-0138-0 (PMC4412034; doi:10.1186/s13045-015-0138-0)

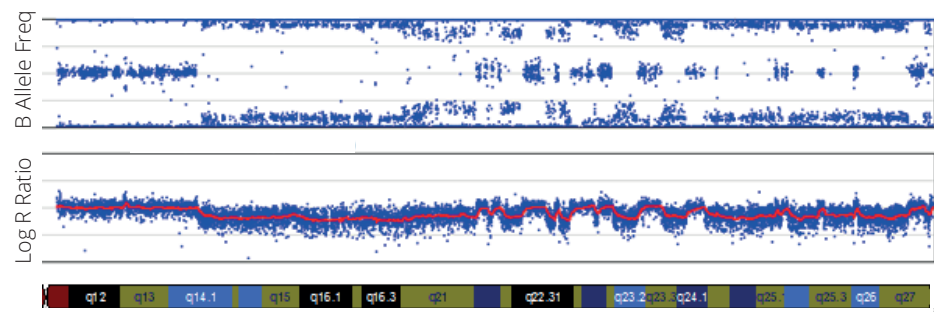

Supplement: Additional file 6: Figure S1. — SNP array analysis results for 6q on case U1. The B allele frequency and log R ratio oscillates between heterozygous/homozygous states and two/one copies, respectively, representing chromothripsis. [file 13045_2015_138_MOESM6_ESM.pdf]
