# Supplementary material for: Deep sequencing and SNP array analyses of pediatric T-cell acute lymphoblastic leukemia reveal NOTCH1 mutations in minor subclones and a high incidence of uniparental isodisomies affecting CDKN2A
Source: J Hematol Oncol. 2015 Apr 24;8:42. doi: 10.1186/s13045-015-0138-0 (PMC4412034; doi:10.1186/s13045-015-0138-0)
Supplement: Additional file 7: Table S6. — Clinical and immunophenotypic features of the 47 T-ALL patients diagnosed in southern Sweden 1983–2011. [file 13045_2015_138_MOESM7_ESM.doc]

**Additional file 7: Table S6.** Clinical and immunophenotypic features of the 47 T-ALL patients diagnosed in southern Sweden 1983-2011

| Case  No. | Sex | Age  (yrs) | WBC count  (x 10^9^/l) | Mediastinal/CNS  involvement | Immunophenotype | Event | Outcome |
| --- | --- | --- | --- | --- | --- | --- | --- |
| 1 | M | 14.8 | 422 | Y/N | CD1-/CD4-/CD8(+)/CD3-/CD7+/CD2+ | N | Alive |
| 2 | F | 10.3 | 28 | N/N | NK | IF | Dead |
| 3 | M | 6.3 | 51 | Y/Y | CD7+/CD4+/CD1-/CD8-/CD3-/CD2- | N | Alive |
| 4 | M | 6.3 | 79 | Y/N | CD7+/CD2+/CD1- | R | Dead |
| 5 | M | 2.9 | 176 | Y/N | CD7+/CD1-/CD5-/CD3-/CD4-/CD8-/CD2- | N | Alive |
| 6 | M | 7.7 | 270 | N/Y | NK | R | Dead |
| 7 | M | 6.5 | 54 | N/N | CD7+/CD2+/CD1- | N | Alive |
| 8 | M | 9.1 | 13 | Y/N | CD7+/CD1(+)/CD5-/CD3-/CD4+/CD8+/CD2- | N | Alive |
| 9 | M | 11.8 | 57 | N/N | CD7+/CD1+/CD5+/CD4+/CD3+/CD8- | N | Alive |
| 10 | M | 16.3 | 290 | Y/N | CD7+/CD2+/CD5+/CD3+/CD34+/CD1-/CD8-/CD4- | R | Dead |
| 11 | M | 10.2 | 24 | N/N | NK | N | Alive |
| 12 | M | 8.4 | 111 | N/N | NK | N | Alive |
| 13 | M | 16.7 | 60 | Y/N | NK | N | Alive |
| 14 | F | 12.3 | 61 | Y/N | CD34+/CD7+/CD2+/CD5+/CD4+/CD8+/CD71+/CD10+/CD3(+) | N | Alive |
| 15 | M | 5.5 | 520 | N/N | CD7+/CD2+/CD5+/CD3+/CD4+/CD8+/CD9+/CD71+ | R | Dead |
| 16 | M | 5.6 | 30 | Y/N | CD45+/CD7+/CD2+/CD1a+/CD5+/CD8+/CD38+/CD3-/HLA-DR-/  CD34-/CD4(+) | N | Alive |
| 17 | M | 11.8 | 307 | Y/N | CD34+/CD7+/CD2+/CD5+/CD3+/HLA-DR(-)/TdT+/CD8-/CD4(+)/CD90- | N | Alive |
| 18 | M | 2.5 | 768 | N/Y | CD3+/CD7+/CD10-/CD19-/CD20-/CD33- | IF | Dead |
| 19 | M | 16.7 | 1.4 | Y/N | CD7+/CD2+/CD5+/CD1a+/CD4+/CD8+/CD3-/CD34-/HLA-DR-/  cCD3+/TdT(+) | N | Alive |
| 20 | M | 3.8 | 73 | Y/N | CD7+/CD2+/CD5+/CD1a+/CD4+/CD8+/CD10+/TdT+/cCD3+/CD3(+) | N | Alive |
| 21 | M | 7.8 | 209 | Y/N | CD3+/CD4+/CD5+/CD2+/CD8+/CD45+/HLA-DR+ | R | Dead |
| 22 | M | 13.9 | 9.3 | Y/N | NK | N | Alive |
| 23 | M | 13.0 | 388 | Y/N | CD34+/CD7+/CD2+/CD5+/CD1a+/CD3+/CD4+/CD8+/TdT+/cCD3(+) | N | Alive |
| 24 | F | 7.0 | 510 | Y/N | CD7+/CD2+/CD5+/CD1a+/CD4+/CD8+/TdT+/CD3-/cCD3+/CD79a(+) | N | Alive |
| 25 | M | 16.8 | 243 | Y/N | CD4+/CD5+/CD8+/CD2+/CD7+/CD34(+)/CD10-/CD19- | DCR1 | Dead |
| 26 | F | 12.9 | 77 | N/N | CD34+/CD117+/CD13+/CD33+/CD10+/CD7+/CD5+/TdT+/  cCD3+/ HLA-DR(+) | DCR1 | Dead |
| 27 | M | 2.6 | 68 | N/N | CD7+/CD2+/CD5+/cCD3+/HLA-DR-/CD34-/CD3-/CD4-/CD8-/  CD1a-/TdT- | N | Alive |
| 28 | M | 13.4 | 12 | Y/Y | CD7+/CD2+/CD5+/CD1a?/CD4+/CD8+/CD117+/CD3(+)/cCD3+ | IF | Dead |
| 29 | M | 9.4 | 54 | Y/N | CD34+/CD45+/CD2+/CD5+/CD7+/CD4+/cCD3+/CD10+/TdT(+) | R | Dead |
| 30 | M | 9.6 | 118 | Y/N | CD3+/CD2+/CD5+/CD7+/CD10+/CD34+/CD4-/CD8-/CD13-/CD33- | N | Alive |
| 31 | M | 4.2 | 12 | Y/N | CD7+/CD2+/CD8+/CD5+/CD1a(+)/cCD3+/CD4-/TdT+/CD3- | N | Alive |
| 32 | M | 4.7 | 72 | Y/N | CD7+/CD2+/CD5+/CD1a+/CD4(+)/CD8+/CD10+/TdT+/cCD3+ | N | Alive |
| 33 | F | 14.7 | 424 | Y/N | CD34+/CD7+/CD2+/CD5+/CD3(+)/TdT+/cCD3+/CD4-/CD8-/CD1a- | IF | Dead |
| 34 | M | 17.2 | 67 | N/N | cCD3+/TdT+/CD2+/CD7+/CD38+/CD13+/CD11b/mCD22(+)/  cCd22-/CD79a-/CD10-/CD20-/CD117- | IF | Alive |
| 35 | M | 7.5 | 187 | N/N | CD7+/CD2+/CD5+/CD1a+/CD4(+)/CD8+/CD10+/TdT+/cCD3+/CD3- | R | Alive |
| 36 | M | 13.1 | 13 | Y/N | CD2+/CD4+/CD5+/CD7+/CD8(+)/cCD3+/CD38+/CD58+/CD99+/  TdT+/mCD3-/CD1a-/CD34- | N | Alive |
| 37 | M | 2.6 | 7.8 | Y/Y | CD7+/CD2-/CD5+/CD1a-/CD3+/CD4-/CD8-/CD56-/CD57-/CD25-/  CD34-/TdT-/CD10- | IF | Alive |
| 38 | M | 2.8 | 391 | N/N | CD7+/CD2+/CD5+/CD1a(+)/CD3-/CD4+/CD8+/CD10+ | N | Alive |
| 39 | M | 16.7 | 218 | N/N | HLA-DR-/CD34+/CD7+/CD2+/CD5+/CD1a(+)/CD3-/TdT+/cCD3+ | N | Alive |
| 40 | F | 1.9 | 134 | Y/NK | mCD3dim+/cCD3+/CD2+/CD5+/CD7+/CD34(+)/CD38+/CD45+/  CD99+/TdT+ | R | Dead |
| 41 | F | 15.8 | 72 | N/NK | HLA-DR-/CD34-/CD7+/CD2+/CD5+/CD3-/CD4+/CD8+/CD1a-/CD99++ | DCR1 | Dead |
| 42 | M | 9.1 | 241 | Y/NK | CD34(+)/CD7+/CD2+/CD5+/CD1a+/CD3-/CD4+/CD8+/CD99++/  CD10(+)/TdT+/cCD3++/CD79a+ | N | Alive |
| 43 | M | 10.0 | 92 | N/NK | CD7+/CD2+/CD5+/CD3-/CD4-/CD8(+)/CD99+/cCD3++/TdT(+) | N | Alive |
| 44 | M | 1.8 | 214 | N/NK | CD7+/CD2+/CD45+/CD99++/CD8++/CD4dim+/TdTdim+/CD1a+/  cCD3+/CD3-/Cd5-/CD10-/CD34-/HLA-DR- | N | Alive |
| 45 | F | 0.7 | 92 | Y/NK | CD7+/CD2-/CD5+/CD1a+/CD4(+)/CD8+/CD99+/cCD3+ | N | Alive |
| 46 | M | 15.0 | 394 | Y/NK | cCD3+/CD5+/CD7+/CD8+/CD2+/CD34+/CD99+/TdT+/CD4-/  mCD3-/CD1a-/HLA-DR-/CD10- | N | Alive |
| 47 | F | 2.0 | 228 | N/NK | CD7+/CD2+/CD5+/CD3-/CD8+/CD4(+)/CD99+/TdT++/cCD3+ | R | Dead |

CNS, central nervous system; DCR1, death in complete remission 1; F, female; IF, induction failure; M, Male; N, no; NK, not known; R, relapse; T-ALL, T-cell acute lymphoblastic leukemia; WBC, white blood cell; Y, yes; yrs, years.
