# Supplementary material for: Deep sequencing and SNP array analyses of pediatric T-cell acute lymphoblastic leukemia reveal NOTCH1 mutations in minor subclones and a high incidence of uniparental isodisomies affecting CDKN2A
Source: J Hematol Oncol. 2015 Apr 24;8:42. doi: 10.1186/s13045-015-0138-0 (PMC4412034; doi:10.1186/s13045-015-0138-0)
Supplement: Additional file 8: Table S7. — The 75 genes sequenced and their chromosomal positions. [file 13045_2015_138_MOESM8_ESM.doc]

**Additional file 8: Table S7.** The 75 genes sequenced and their chromosomal positions

| Gene symbol | Gene name | Position^a^ |
| --- | --- | --- |
| *AKT1* | v-akt murine thymoma viral oncogene homolog 1 | 14q32.33 |
| *BCL11B* | B-cell CLL/lymphoma 11B (zinc finger protein) | 14q32.2 |
| *BRAF* | v-raf murine sarcoma viral oncogene homolog B1 | 7q34 |
| *BTG1* | B-cell translocation gene 1, anti-proliferative | 12q21.33 |
| *CCND2* | cyclin D2 | 12p13.32 |
| *CDKN2A* | cyclin-dependent kinase inhibitor 2A | 9p21.3 |
| *CDKN2B* | cyclin-dependent kinase inhibitor 2B | 9p21.3 |
| *CDKN2C* | cyclin-dependent kinase inhibitor 2C | 1p32.3 |
| *CREBBP* | CREB binding protein | 16p13.3 |
| *CTCF* | CCCTC-binding factor (zinc finger protein) | 16q22.1 |
| *DCLRE1C* | DNA cross-link repair 1C | 10p13 |
| *DNM2* | dynamin 2 | 19p13.2 |
| *DNMT3A* | DNA (cytosine-5-)-methyltransferase 3 alpha | 2p23.3 |
| *ECT2L* | epithelial cell transforming sequence 2 oncogene-like | 6q24.1 |
| *EED* | embryonic ectoderm development | 11q14.2 |
| *ENPEP* | glutamyl aminopeptidase (aminopeptidase A) | 4q25 |
| *EP300* | E1A binding protein p300 | 22q13.2 |
| *ETV6* | ets variant 6 | 12p13.2 |
| *EZH2* | enhancer of zeste homolog 2 (Drosophila) | 7q36.1 |
| *FBXW7* | F-box and WD repeat domain containing 7, E3 ubiquitin protein ligase | 4q31.3 |
| *FGFR1* | fibroblast growth factor receptor 1 | 8p11.22-11.23 |
| *FLT3* | fms-related tyrosine kinase 3 | 13q12.2 |
| *GATA3* | GATA binding protein 3 | 10p14 |
| *GFI1* | growth factor independent 1 transcription repressor | 1p22.1 |
| *HIST1H1B* | histone cluster 1, H1b | 6p22.1 |
| *HNF1A* | HNF1 homeobox A | 12q24.31 |
| *HNRNPA1* | heterogeneous nuclear ribonucleoprotein A1 | 12q13.13 |
| *HNRNPR* | heterogeneous nuclear ribonucleoprotein R | 1p36.12 |
| *HOXA10* | homeobox A10 | 7p15.2 |
| *HOXA11* | homeobox A11 | 7p15.2 |
| *IDH1* | isocitrate dehydrogenase 1 (NADP+), soluble | 2q34 |
| *IDH2* | isocitrate dehydrogenase 2 (NADP+), mitochondrial | 15q26.1 |
| *IL7R* | interleukin 7 receptor | 5p13.2 |
| *IKZF1* | IKAROS family zinc finger 1 (Ikaros) | 7p12.2 |
| *IRS4* | insulin receptor substrate 4 | Xq22.3 |
| *JAK1* | Janus kinase 1 | 1p31.3 |
| *JAK2* | Janus kinase 2 | 9p24.1 |
| *JAK3* | Janus kinase 3 | 19p13.11 |
| *KRAS* | v-Ki-ras2 Kirsten rat sarcoma viral oncogene homolog | 12p12.1 |
| *LCK* | lymphocyte-specific protein tyrosine kinase | 1p35.1 |
| *LEF1* | lymphoid enhancer-binding factor 1 | 4q25 |
| *LMO1* | LIM domain only 1 (rhombotin 1) | 11p15.4 |
| *LMO2* | LIM domain only 2 (rhombotin-like 1) | 11p13 |
| *LYL1* | lymphoblastic leukemia derived sequence 1 | 19p13.2 |
| *MTOR* | mechanistic target of rapamycin (serine/threonine kinase) | 1p36.22 |
| *MYB* | v-myb myeloblastosis viral oncogene homolog (avian) | 6q23.3 |
| *NF1* | neurofibromin 1 | 17q11.2 |
| *NOTCH1* | notch 1 | 9q34.3 |
| *NRAS* | neuroblastoma RAS viral (v-ras) oncogene homolog | 1p13.2 |
| *OLIG2* | oligodendrocyte lineage transcription factor 2 | 21q22.11 |
| *PAX5* | paired box 5 | 9p13.2 |
| *PHF6* | PHD finger protein 6 | Xq26.2 |
| *PIK3CA* | phosphatidylinositol-4,5-bisphosphate 3-kinase, catalytic subunit alpha | 3q26.32 |
| *PTEN* | phosphatase and tensin homolog | 10q23.31 |
| *PTPN2* | protein tyrosine phosphatase, non-receptor type 2 | 18p11.21 |
| *PTPN11* | protein tyrosine phosphatase, non-receptor type 11 | 12q24.13 |
| *PTPRC* | protein tyrosine phosphatase, receptor type, C | 1q31.3-32.1 |
| *RANBP17* | RAN binding protein 17 | 5q35.1 |
| *RB1* | retinoblastoma 1 | 13q14.2 |
| *RBPJ* | recombination signal binding protein for immunoglobulin kappa J region | 4p15.2 |
| *RELN* | reelin | 7q22.1 |
| *RUNX1* | runt-related transcription factor 1 | 21q22.12 |
| *SETD2* | SET domain containing 2 | 3p21.31 |
| *SH2B3* | SH2B adaptor protein 3 | 12q24.12 |
| *SPI1* | spleen focus forming virus (SFFV) proviral integration oncogene spi1 | 11p11.2 |
| *SUZ12* | suppressor of zeste 12 homolog (Drosophila) | 17q11.2 |
| *TAL1* | T-cell acute lymphocytic leukemia 1 | 1p33 |
| *TAL2* | T-cell acute lymphocytic leukemia 2 | 9q31.2 |
| *TCF3* | transcription factor 3 | 19p13.3 |
| *TCF12* | transcription factor 12 | 15q21.3 |
| *TET2* | tet methylcytosine dioxygenase 2 | 4q24 |
| *TLX1* | T-cell leukemia homeobox 1 | 10q24.31 |
| *TLX3* | T-cell leukemia homeobox 3 | 5q35.1 |
| *TP53* | tumor protein p53 | 17p13.1 |
| *WT1* | Wilms tumor 1 | 11p13 |

^a^According to Ensembl release 75, http://www.ensembl.org.
